# Genetic and multi-omic resources for Alzheimer disease and related dementia from the Knight Alzheimer Disease Research Center

**DOI:** 10.1038/s41597-024-03485-9

**Published:** 2024-07-12

**Authors:** Maria Victoria Fernandez, Menghan Liu, Aleksandra Beric, Matt Johnson, Arda Cetin, Maulik Patel, John Budde, Pat Kohlfeld, Kristy Bergmann, Joseph Lowery, Allison Flynn, William Brock, Brenda Sanchez Montejo, Jen Gentsch, Nicholas Sykora, Joanne Norton, Jen Gentsch, Olga Valdez, Priyanka Gorijala, Jessie Sanford, Yichen Sun, Ciyang Wang, Dan Western, Jigyasha Timsina, Tassia Mangetti Goncalves, Anh N. Do, Yun Ju Sung, Guoyan Zhao, John C. Morris, Krista Moulder, David M. Holtzman, Randall J. Bateman, Celeste Karch, Jason Hassenstab, Chengjie Xiong, Suzanne E. Schindler, Joyce (Joy) Balls-Berry, Tammie L. S. Benzinger, Richard J. Perrin, Andrea Denny, B. Joy Snider, Susan L. Stark, Laura Ibanez, Carlos Cruchaga

**Affiliations:** 1 Department of Psychiatry, Washington University School of Medicine, St. Louis, MO USA; 2 NeuroGenomics and Informatics Center, Washington University School of Medicine, St. Louis, MO 63110 USA; 3 Research Center and Memory Clinic, ACE Alzheimer Center, Barcelona, Spain; 4 Department of Genetics, Washington University School of Medicine, St. Louis, MO USA; 5 Division of Biostatistics, Washington University School of Medicine, St. Louis, MO 63110 USA; 6 Department of Neurology, Washington University School of Medicine, St. Louis, MO 63110 USA; 7 Pathology and Immunology Department, Washington University School of Medicine, St. Louis, MO 63110 USA; 8 Knight Alzheimer Disease Research Center, Washington University School of Medicine, St. Louis, MO USA; 9 Hope Center for Neurological Disorders, Washington University School of Medicine, St. Louis, MO USA; 10 Dominantly Inherited Alzheimer Disease Network (DIAN), St. Louis, USA; 11 Radiology Department, Washington University School of Medicine, St. Louis, MO 63110 USA; 12 Occupational Therapy, Neurology and Social Work, St. Louis, USA

**Keywords:** Genetic databases, Diagnostic markers, Alzheimer's disease

## Abstract

The Knight-Alzheimer Disease Research Center (Knight-ADRC) at Washington University in St. Louis has pioneered and led worldwide seminal studies that have expanded our clinical, social, pathological, and molecular understanding of Alzheimer Disease. Over more than 40 years, research volunteers have been recruited to participate in cognitive, neuropsychologic, imaging, fluid biomarkers, genomic and multi-omic studies. Tissue and longitudinal data collected to foster, facilitate, and support research on dementia and aging. The Genetics and high throughput -*omics* core (GHTO) have collected of more than 26,000 biological samples from 6,625 Knight-ADRC participants. Samples available include longitudinal DNA, RNA, non-fasted plasma, cerebrospinal fluid pellets, and peripheral blood mononuclear cells. The GHTO has performed deep molecular profiling (genomic, transcriptomic, epigenomic, proteomic, and metabolomic) from large number of brain (n = 2,117), CSF (n = 2,012) and blood/plasma (n = 8,265) samples with the goal of identifying novel risk and protective variants, identify novel molecular biomarkers and causal and druggable targets. Overall, the resources available at GHTO support the increase of our understanding of Alzheimer Disease.

## Background & Summary

Alzheimer disease (AD) currently affects approximately 7 million Americans, and it is projected to double by 2050^1^. AD is pathologically characterized by the extracellular accumulation of misfolded amyloid-beta protein, and intraneuronal accumulation of aggregates of hyperphosphorylated tau. This causes neuronal death, inflammation, brain atrophy among many other deleterious changes^2^. These molecular events manifest with a progressive memory and cognitive decline. There are two types of AD, sporadic and Autosomal Dominant (ADAD)^3^. The exact cause of sporadic AD is not fully understood, with genetic and environmental causes associated. ADAD is caused by mutations or duplications of the amyloid precursor protein (*APP*), mutations in presenilin 1 (*PSEN1*), or mutations in presenilin 2 (*PSEN2*), that present in an autosomal dominant inheritance pattern with symptom onset before 65 years old^4,5^. Regardless of cause, research to identify biomarkers that can aid in the diagnosis, especially before symptom onset, and the approval of a disease modifying therapy have been the main drivers of the research field.

The Knight Alzheimer Disease Research Center (Knight-ADRC) was stablished in 1985, and it is one of the 33 Alzheimer Disease Research Centers (ADRCs) funded by the National Institute on Aging. ADRCs are centers of excellence located at major medical institutions across the United States (https://www.nia.nih.gov/health/alzheimers-disease-research-centers). However, aging research at Washington University in St. Louis (WUSTL) dates from 1979, with the initiation of the Memory and Aging Project (MAP)^6^. The MAP facilitated the establishment of three major projects that have been continuously funded by the National Institute on Aging of the NIH (NIA): (i) Healthy Aging and Senile Dementia (HASD) firstly awarded in 1984 [P01 AG003991]; (ii) the Alzheimer Disease Research Center (ADRC), funded in 1985 [P30 AG066444]; and (iii) the Adult Children Study (ACS)^7^ funded in 2005 [P01 AG026276]. The ACS main goal is to study preclinical AD, including familial forms in the context of the Familial Adult Children Study, that was focused on studying the offspring of individuals afflicted with AD due to the presence of a causative mutation. This study led to the establishment of the Dominantly Inherited Alzheimer Network (DIAN) in 2008 [U19 AG032438]^8^. More recently, in 2010, Charles F. Knight and his wife, Joanne Knight endowed the ADRC, becoming the Charles F. and Joanne Knight Alzheimer Disease Research Center (Knight-ADRC).

For more than 40 years, the Knight-ADRC team at WUSTL has studied cognitive functioning in persons as they age. Volunteers and participants of the Knight-ADRC must meet three inclusion criteria: (i) be 40 years or older; (ii) present with stable general health; and (iii) having no memory or thinking problems, or very mild or mild dementia. Individuals recruited by the Knight-ADRC that are found to carry a causal mutation in *APP, PSEN1*, or *PSEN2* are referred to The Dominantly Inherited Alzheimer Network (DIAN) study. DIAN facilitates the study of the natural history of the individuals and families with autosomal dominant AD, with special focus in the critical preclinical period. The DIAN participants are out of the scope of the present manuscript.

All participants recruited by the Knight-ADRC undertake annual memory and thinking assessments if they are older than 65 years of age, or every three years if younger. Blood samples and CSF are collected every other year. Magnetic Resonance Imaging (MRI) along with amyloid and tau positron emission tomography (PET) scans are also performed every two years and they can be requested by qualified investigators. The Knight-ADRC implemented the Uniform Data Set^9^, which allows harmonization across all three projects (MAP, HASD, and ACS) and facilitates data and sample sharing with qualified investigators. Finally, the Knight-ADRC contributes data to the following genetic and genomic NIH funded initiatives: National Alzheimer Coordinating Center (NCRAD), Alzheimer Disease Genetics Consortium (ADGC), National Institute on Aging Genetics of Alzheimer’s Disease Data Storage (NIAGADs), Alzheimer Disease Neuroimaging Initiative (ADNI), Alzheimer’s Disease Sequencing Project (ADSP), Global Neurodegeneration Proteomics Consortium (GNPC), and the Alzheimer Clinical Trials Consortium (ACTC).

**Fig. 1:**
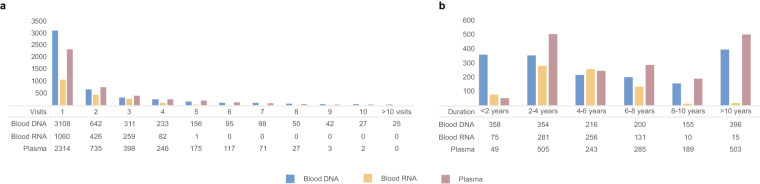
Longitudinal blood DNA, RNA, and unfasted plasma samples stored at the GHTO (**a**). Stored samples at GHTO by number of assessments of each Knight-ADRC participant (**b**). Time between assessments with phlebotomy for Knight-ADRC participants with more than one visit. Blue represents DNA isolated from blood, yellow RNA extracted from blood, and dark pink for unfasted plasma.

Over the years, the Knight-ADRC has performed cognitive assessments to more than 6,625 participants, with some participants active for over 30 years (Fig. 1a,b). Around 1,182 participants have donated their brains for autopsy and neuropathological examination, several of them with some level of omic data available (n = 817). Of all participants, DNA is available for 4,787 participants, RNA from 1,828 participants, fasted CSF and plasma samples for more than 1,240 participants (Fig. 1a).

The **Genetics & High Throughput – Omics Core** (GHTO) is located at the NeuroGenomics and Informatics (NGI) Center at WUSTL, and it is one of the eleven cores that comprises the Knight-ADRC. It acts as biologic and data repository for DNA, RNA, peripheral blood mononuclear cells (PBMCs), nonfasted plasma, and CSF cell pellets collected from Knight-ADRC participants. All samples are genotyped for *APOE* allele determination and for known causal AD mutation. It also acts as *-omic* data repository for genomic (GWAS, whole exome, and whole genome sequencing), transcriptomic (coding and non-coding transcriptome, including small, long-noncoding or circRNAs and spatial transcriptomics), epigenomics, proteomic, lipidomic, and metabolomic data. To date, more than 20,500 biological samples (whole blood, brain, plasma, and CSF) from 5,283 Knight-ADRC participants have already been used to generate some or several layers of omic data. To ensure quality, integrity, and compatibility of the generated data, the GHTO Core harmonizes all the molecular datasets. Additionally, the samples are linked to the clinical, imaging, biomarker and neuropathological data. In summary, the GHTO Core integrates harmonized phenotypic data with harmonized *-omic* data-layers to produce deep molecular profiling of Knight-ADRC participants that (i) provides QCed, harmonized and analyses ready molecular dataset for Knight-ADRC participants^10–14^, (ii) facilitates research for novel risk factors^15–18^, (iii) enhances identification of disease biomarkers^15,19^, and (iv) promotes the generation of disease predictors^10,20^.

## Methods

### Demographics of Knight-ADRC participants with *-omic* data

The Institutional Review Board (IRB) of Washington University School of Medicine in St. Louis approved the study with the IRB number 201109148, and research was performed in accordance with the approved protocols. Participants are asked to sign the informed consent by the staff at the Knight-ADRC. To allow for family member recruitment, interested participants are consented in-person during community events, while others are consented over the phone followed by the shipment of two paper copies of the consent. One copy will be signed and sent back to the Knight-ADRC, and the other will be retained by the participant. Individuals with memory issues must have a Legally Authorized Representative present at the time of enrollment. When no legal guardian or attorney-in-fact is present, a spouse, adult child, parent, sibling, or relative may sign.

Knight-ADRC participants must be at least 40 years old and have no memory problems or, at the most, mild dementia at the time of enrollment. They are recruited thought the Memory and Aging Project and Memory Diagnostic Clinic participant pool. Flyers, word of mouth, and referrals are the main tools for participants to be enrolled at the Knight-ADRC. To characterize participants the Knight-ADRC uses a combination of clinical, psychometric, biochemical, and imaging information. Clinical diagnosis is performed by neurologist at the clinical core and use Clinical Dementia Rating^®^ (CDR^®^) to determine the memory impairment. A CDR^®^ score 0.5 corresponds to very mild dementia, 1 to mild dementia whereas a CDR^®^ = 0 represent no cognitive impairment. This classification is re-evaluated for all participants at every assessment. At death, autopsy will provide a neuropathological diagnosis even though brain donation is not requirement for enrollment. To date, 1,182 participants have donated their brains and are stored at the Knight-ADRC Neuropathological core.

Out of the 6,625 Knight-ADRC participants (Table 1) recruited during the last 30 years, 2,746 of them are classified at the last assessment as AD (with 29 of them being Autosomal Dominant AD), 2,369 are considered cognitively unimpaired, and 1,510 are considered to suffer from other dementias (1,285 AD Related Dementias (ADRD), 153 FTD, and 72 DLB - Table 1. Overall, most of the participants self-report as European Americans or Non-Hispanic Whites (NHW, 83.6%) and African American (AA, 12.3%); the remaining 4.1% self-report as Hispanic White (HW), Asian, American Indian, Alaskan Native, Native Hawaiian, and Pacific Islander (Table 1). The cohort is almost even regarding sex, with 57.3% identifying as females (Table 1). However, within the AA participants, females have a higher representation (73%) compared to the NHW (56%). As reported in previous studies, different *APOE* genotype distribution is observed between ethnic groups (Table 2).

**Table 1:** Basic demographics of Knight-ADRC participants by disease status at last assessment.

	AD	ADAD	ADRD	Control	DLB	FTD	Total
**Sample Size**	2,717	29	1285	2,369	72	153	6,625
**% Female**	60.36	41.38	48.25	60.11	45.83	43.14	57.28
**% APOE4+**	57.94	65.22	44.35	33.47	41.18	43.36	45.64
**Age at onset**	72.93	58.58	71.3	.	71.46	64.69	72.25
**Age at last assessment**	80.18	62.3	77.39	72.45	78.97	66.72	77
**Self-Reported Ethnicity [n,(%)]**							
**African American**	329 (40.5%)	.	104 (12.8%)	376 46.3%)	3 (0.4%)	.	812 (12.3%)
**American Indian or Alaska Native**	8 (53.3%)	.	1 (6.7%)	4 (26.7%)	2 (13.3%)	.	15 (0.2%)
**Asian**	8 (44.4%)	.	2 (11.1%)	8 (44.4%)	.	.	18 (0.3%)
**Native Hawaiian or Other Pacific Islander**	.	.	.	2 (100%)	.	.	2 (0.03%)
**Non-Hispanic White**	2,328 (42.0%)	28 (0.5%)	1,009 (18.2%)	1,962 (35.4%)	62 1.1%	150 (2.7%)	5,539 (83.6%)
**Hispanic White**	6 (37.5%)	.	5 (31.3%)	4 (25.0%)	.	1 (6.3%)	16 (0.2%)
**More than one race**	3 (60.0%)	.	.	2 (40.0%)	.	.	5 (0.08%)
**Not-Reported**	35 (16.1%)	1 (0.5%)	164 (75.2%)	11 (5.0%)	5 (2.3%)	2 (0.9%)	218 (3.3%)

AD = Alzheimer Disease; ADAD = Autosomal Dominant Alzheimer Disease; ADRD = Alzheimer Disease Related Dementia; DLB = Lewy Body Dementia; FTD = FrontoTemporal Dementia.

**Table 2:** *APOE* Genotype differences across self-reported ethnicity from the participants of the Knight-ADRC.

Self-Reported Ethnicity	*APOE* genotype					
	22	23	24	33	34	44
**African American**	10 (1.4%)	66 (9.2%)	27 (3.8%)	305 (42.7%)	260 (36.4%)	47 (6.6%)
**American Indian or Alaska Native**	.	3 (21.4%)	.	6 (42.9%)	3 (21.4%)	2 (14.3%)
**Asian**	.	3 (17.6%)	.	7 (41.2%)	5 (29.4%)	2 (11.8%)
**Native Hawaiian or Other Pacific Islander**	.	.	.	1 (50%)	1 (50%)	.
**Non-Hispanic White**	15 (0.3%)	411 (8.4%)	148 (3.0%)	2233 (45.9%)	1718 (35.3%)	344 (7.1%)
**Hispanic White**	.	.	.	8 (57.1%)	4 (28.6%)	2 (14.3%)
**More than one race**	.	.	.	3 (75.0%)	.	1 (25%)
**Not Reported**	.	6 (6.8%)	4 (4.5%)	34 (38.6%)	33 (37.5%)	11 (12.5%)

### Resources of Knight-ADRC participants

The GHTO Core leverages the resources from the Knight-ADRC to further understand the pathobiology of AD. It aims to identify novel risk and protective molecular factors along with biomarkers for AD. To do so, the GHTO relies on access to high quality biological samples and accurate and detailed phenotypes. The GHTO stores nucleic acids (DNA and RNA) extracted mainly from blood, but also brain, CSF, and plasma. To date, the GHTO has banked more than 9,633 blood DNA samples from 4,787 unique participants (Table 3). DNA and RNA has been extracted from 1,178 brain samples (817 unique participants – Tables 4), and 3,022 blood RNA samples from 1,828 unique participants (Table 3). Recently, the GHTO core initiated the isolation and storage of PMBCs (675 samples from 609 participants) as well as the storage of CSF cell pellets (252 samples from 250 participants). The CSF and brain samples are not stored long term at the GHTO, they are obtained via collaboration with the Liquid Biomarker and the Neuropathological Knight-ADRC cores respectively. Finally, GHTO also collects and banks non-fasted plasmas (8,299 samples from 4,088 participants; Table 3; Fig. 1a). There are 1,701 participants that have donated plasma more than once, with some of them with up to nine longitudinal plasma samples spanning 12 years (Fig. 1a,b). Similarly, DNA and RNA have been extracted from individuals at different time-points. DNA is available for a total of 5,269 visits, having up to 14 visits from some participants and spanning more than 10 years (Fig. 1a,b). Similarly, RNA is available for 1,831 blood samples, with some participants with up to 5 visits (Fig. 1a,b).

**Table 3:** Samples currently stored at the GHTO.

	Total Visits Blood Collection		Unique Participants Blood Collection		PBMC		CSF*			Plasma	
	DNA	RNA	DNA	RNA	All	Unique IDs	Cell Pellet	All	Unique IDs	All	Unique IDs
**AD**	2,821	686	1,648	423	94	88	28	519	401	2,809	1534
**ADAD**	42	7	16	3	1	1	.	14	5	31	11
**ADRD**	1,261	576	924	510	84	84	46	136	96	1,230	827
**Control**	5,275	1,700	2,045	849	494	434	178	1,425	725	4,087	1,622
**DLB**	58	22	40	20	.	.	.	4	4	59	40
**FTD**	176	31	114	23	2	2	.	12	9	83	54
**Total**	9,633	3,022	4,787	1,828	675	609	252	2,110	1,240	8,299	4,088

*Residual samples used to generate omic data obtained via tissue request. Knight-ADRC CSF biobanking is performed by the Fluid Biomarker Core.

PBMC = Peripheral Blood Mononuclear Cell; CSF = CerebroSpinal Fluid; DNA = DesoxyriboNucleic Acid; RNA = RiboNucleic Acid; AD = Alzheimer Disease; ADAD = Autosomal Dominant Alzheimer Disease; ADRD = Alzheimer Disease Related Dementia; DLB = Lewy Body Dementia; FTD = FrontoTemporal Dementia

Each sample type includes total samples and unique individual to account for the longitudinal biological sampling. Brain sample information can be found in Table 4.

**Table 4:** Brain samples currently at the GHTO distributed by brain region.

	Cerebellum	Frontal Cortex	Parietal	Temporal Cortex	Total
**AD**	451	28	344	17	840
**ADAD**	7		7		14
**ADRD**	91	1	34	1	127
**Control**	44	8	35	4	91
**DLB**	28	2	14	2	46
**FTD**	34	3	20	3	60
**Total**	655	42	454*	27	**1,178**

*Current samples available at GHTO. Mismatch with samples with –omic data due to sample depletion. AD = Alzheimer Disease; ADAD = Autosomal Dominant Alzheimer Disease; ADRD = Alzheimer Disease Related Dementia; DLB = Lewy Body Dementia; FTD = FrontoTemporal Dementia

Most of them have already been used to generate high-throughput data. Brain tissue was accessed via tissue request to the Neuropathological Core from the Knight-ADRC.

**Fig. 2:**
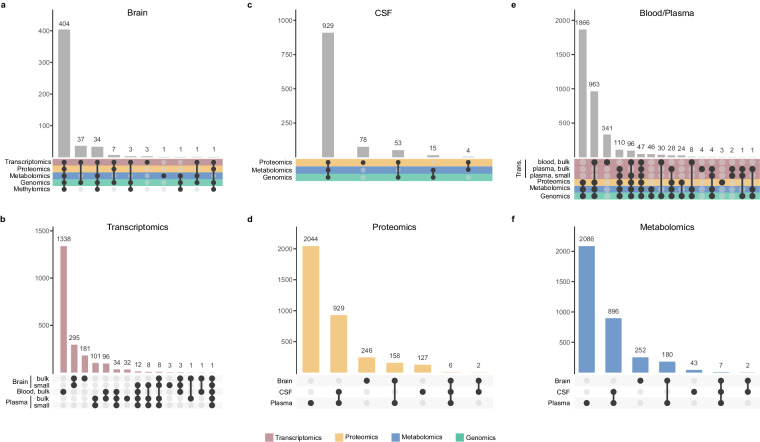
Upset plots summarizing the *-omic* data layers generated by GHTO investigators and available to the scientific community. This representation does not contain longitudinal samples or brain regions, all graphs represent unique individuals with at least one data point (**a**). Summary of all the high-throughput data available for brain samples; (**b**). Distribution of the transcriptomic data by RNA tissue of origin; (**c**). Fasted CSF proteomic and metabolomic data available; (**d**). High-throughput protein measurements performed across tissues (brain, CSF, and plasma); (**e**). High-throughput data availability from whole blood (bulk transcriptomics) and plasma; and (**f**). Metabolomic data generated in brain, CSF, and plasma samples. Transcriptomic data is depicted with dark pink, proteomic data in yellow, metabolomic data in blue, and genomic data in green.

All these resources are being leveraged to generate state of the art high throughput data. As of October 2023, *-omic* data from more than 26,000 biological samples (blood, several brain regions, plasma, and CSF) has been generated, from 5,283 Knight-ADRC participants (Table 5). Genomic data has been generated for the 5,283 Knight-ADRC participants: 4,843 genome wide array data (GWAs); 1,069 whole exome sequencing (WES), and 2,074 whole genome sequencing (WGS). Transcriptomic data (bulk RNA-Seq, ribodepletion) is available for 2,334 whole blood samples from 1,485 unique participants, and 630 brain samples from 490 unique participants. Additionally, small RNA sequencing is available for 330 parietal brains samples and 164 plasma samples (Fig. 2a,b). Finally, plasma cell-free RNA (cfRNA) sequence data is also available for 293 participants. High throughput proteomic data is available for 412 brain samples (Fig. 2a), 1,159 CSF samples (1,064 unique participants – Fig. 2c,d), and 4,084 plasma samples (3,137 unique participants – Fig. 2e). Metabolomics and lipidomic has been generated from 455 brain, 948 CSF and 3,169 plasma samples (Fig. 2f). Additionally, methylation data from 464 brain samples (from 444 unique individuals) and 20 blood samples is also available. As shown in Fig. 2, there is a high level of overlap, with multiple Knight-ADRC participants with multiple data layers available. In summary, blood transcriptomic, proteomic, metabolomic, and lipidomic data is available for 1,106 participants and brain transcriptomic, proteomic, methylomic, and metabolomic data is available for 404 participants.

**Table 5:** Knight-ADRC participants with high-throughput data available distributed by data type and tissue of origin.

	Genomics			Proteomics			Metabolomics & Lipidomics			Methylation		Transcriptomics				
	Array-based	WES	WGS	Plasma	CSF	Brain	Plasma	CSF	Brain	Brain	Blood	Brain sRNA	Plasma sRNA	Plasma cfRNA	Blood	Brain
**AD**	2,085	578	928	1,266	295	320	1,285	286	342	344	15	283	94	134	312	371
**ADAD**	20	5	16	10	4	6	9	4	7	7	.	4	.	.	2	7
**ADRD**	531	85	165	349	109	32	359	69	33	34	.	3	17	21	359	38
**Control**	1,999	382	907	1,432	642	27	1,431	575	29	29	5	34	29	110	801	37
**DLB**	64	10	20	35	4	9	37	4	11	11	.	3	13	16	2	14
**FTD**	144	9	38	45	10	18	48	10	19	19	.	3	11	12	9	23
**Total**	4,843	1,069	2,074	3,137	1,064	412	3,169	948	441	444	20	330	164	293	1,485	490

WES = Whole Exome Sequencing; WGS = Whole Genome Sequencing; CSF = CerebroSpinal Fluid; sRNA = Small RNA; cfRNA = Cell-Free RNA; AD = Alzheimer Disease; ADAD = Autosomal Dominant Alzheimer Disease; ADRD = Alzheimer Disease Related Dementia; DLB = Lewy Body Dementia; FTD = FrontoTemporal Dementia

This table does not include longitudinal sample; for several individuals, more than one time-point (or brain region) is available.

### Genomic data

#### Overview of genomic data: Array, WES and WGS data

**Fig. 3:**
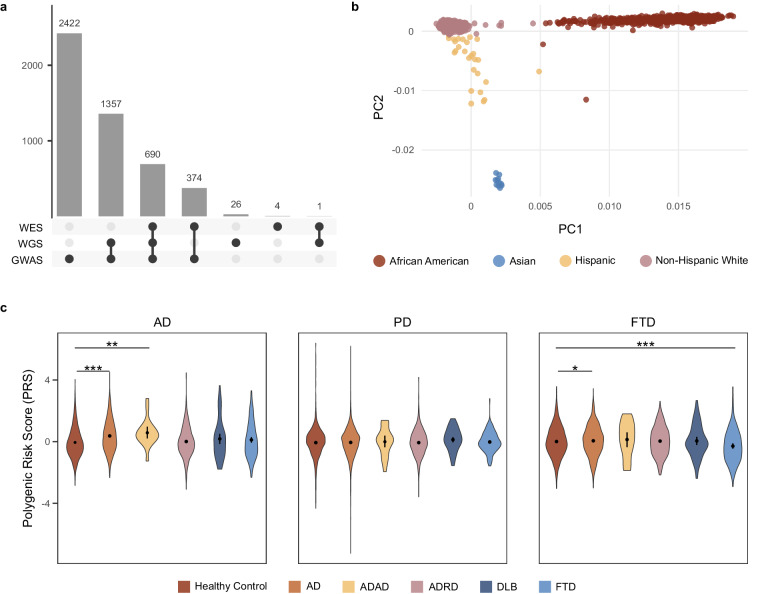
Genomic data available at the GHTO (**a**). Distribution of genomic data from Knight-ADRC individuals across technologies (**b**). Ancestry distribution of Knight-ADRC participants with genomic data available calculated using the first two principal components (**c**). Polygenic risk scores for AD, PD, and FTD distribution across knight-ADRC participants by diagnostic at last assessment. WES = Whole Exome Sequencing; WGS = Whole Genome Sequencing; GWAS = Genome Wide Association Study; AD = Alzheimer Disease; ADAD = Autosomal Dominant Alzheimer Disease; ADRD = Alzheimer Disease Related Dementia; DLB = Lewy Body Dementia; FTD = FrontoTemporal Dementia.

Over the years, three types of genomic data have been generated for the Knight-ADRC samples: array-based genotyping, Whole Exome Sequence (WES), and Whole Genome Sequence (WGS). There are 4,843 participants with array-based genotyping data, 1,069 with WES completed, and 2,074 with WGS (Table 5). Array-based genotyping and either WES/WGS is available for 2,421 participants (Fig. 3a).

Array-based genotyping has been generated since 2008 using nine chips (Illumina Human660W-Quad, Infinium OmniExpressExome-8, Illumina Omni1-Quad, Illumina Human1M-Duo, Infinium Neuro Consortium Array, Infinium CoreExome-24, Infinium Global Screening Array-24, Human610-Quad, and Affy UK Biobank Axiom). Similarly, whole exome data (WES) data generation started in 2010 using Nimblegen VCRome v2.1 with IDT spike-in, then Agilent human Exon V5 (50 Mb), VCR_crome, and later on Sure Select human all exon 50 Mb kit. Finally, Whole Genome Sequence (WGS) has been generated recently using the Kapa Hyper PCR free using Illumina NovaSeq 6000, while in the past, Illumina HiSeq 4000 or HiSeqX were used. To integrate and analyze all the platforms and data generation approaches, ensuring its homogeneity, the GHTO scientists have established stringent pipelines to generate the genotyping clusters and impute for array-based genotyping, or align and call for WES and WGS, and then perform quality control to finally integrate all genomic data. Please see GitHub (https://github.com/NeuroGenomicsAndInformatics) and NGI website (https://neurogenomics.wustl.edu/open-science) for further details.

#### Array-based genotyping data

##### Sample preparation

The Hope Center DNA/RNA Purification Core at Washington University in Saint Louis (associated to the GHTO) uses the Autogen FlexSTAR^+^ salt precipitation to isolate pure DNA. The FlexSTAR is fully automated to perform high-quality DNA isolation from large volumes (5–10 ml) of whole blood and buffy coat samples. To isolate DNA from small volume samples (<1 ml), that can originate from a range or sources (blood, buffy coat, saliva, blood cards, tissue, buccal swab, plasma, and CSF among others) the Core uses a bead-based automatized purification system, the Maxwell 48 automated workstation. After extraction, DNA quality and quantity are assessed with the TapeStation 4200, a high-throughput automated electrophoresis platform, that runs up to 96 samples in one run. All samples are then normalized to 100 ng/μL and stored in 2D barcoded tubes at −80 °C.

##### Data processing and quality contry

Array-based genotyping data generation, quality control (QC), and imputation is handled separately for each genotyping round. Once the data reaches the GHTO standard of quality, the genotyping rounds are merged. Briefly, after genotyping, all single nucleotide variants (SNPs) are called using Genome Studio. A two-step QC pipeline is implemented to ensure maximum retention of individuals and variants. Firstly, the GHTO uses a loose QC in which all variants and individuals with call rate less than 80% are removed in that order. Then a more stringent second pass using 98% for both parameters prior to data export into plink format. The Plink files are used to feed the TOPMed Imputation Server pipeline using the reference genome GRCh38. After imputation, any variant with an imputation quality of Rsq > 0.30 is retained. For the remaining variants, standard QC is applied: retention of variants and individuals with 98% call rate, removal of SNPs not in Hardy-Weinberg equilibrium (HWE), concordance check between reported sex and genetic sex, and concordance with expected identity-by-descent (IBD) estimates for technical replicates and family members (if present).

Once the different data generation rounds are merged, a final round of stringent QC is applied. Briefly, variants and individuals are filtered by 98% call rate, and autosomal SNPs not in HWE (P < × 10^−6^) are filtered out. The concordance between reported and genetic sex is assessed a second time, any additional discordances are removed. Finally, IBD estimates allows to confirm expected duplicated samples, familial relatedness, and remove any potential sample swap. All the quality control procedures are performed using Plink1.9 or Plink2.0^21^. Full QC report can be found in Appendix 1.

##### Data availability

The Knight-ADRC GHTO has fully imputed and QCd array-based genotyping data available for 4,843 participants which includes 2,085 sporadic AD cases and 1,999 controls (Table 5). This data is available at the NIAGADS Knight-ADRC collection and specifically the NG00127 dataset. Ethnically, 4,173 are NHW, 626 AA, 33 Admixed American, and 11 of Asian genetic background, as determined by genetic principal components (Fig. 3b). Approximately 30,000,000 high quality variants passed QCed and are present in the fully imputed file. At a 98% call rate, 7,202,789 common variants (minor allele frequency (MAF) greater than 5%) and 8,448,089 rare variants (MAF < 1%) are currently available.

#### Genomic data (WES & WGS)

##### Sample preparation

DNA isolation protocols are the same regardless of the genomic data generated downstream. Protocol is described in the Array-based genotyping data section.

##### Data processing and quality control

All original files, regardless of being originated by WES or WGS, or their format (fastq, cram, bam, or sra) are aligned individually to the reference genome GRCh38 following GATK’s Best Practices Workflow standards^22^ with NVIDIA’s Clara Parabricks optimizations for High Performance Computing^23^. The GHTO most current pipeline can be found on GitHub (NeuroGenomicsAndInformatics/WXS-Pipelines (github.com)). Briefly, three different implementations have been generated to accept all different types of incoming files (fasq, alignment BAM or CRAM). The pipeline includes an optimized set of commands to go from sequencing reads to gVCF files. For each input file, the pipeline generates: an aligned file, a sorted file, a duplicate-marked cram file and a gVCF file. Additionally, it will provide a set of QC metrics gathered with GATK’s CollectWgsMetrics, VariantCallingMetrics, and VerifybamID2 to perform a first QC step before joint-calling. Samples with freemix value greater than 0.05 or coverage less than 30x (for WES) or 20x (for WGS) are marked as failed.

Joint Calling is performed by chromosome using GenomicsDBImport for WES and GenotypeGVCFs for WGS. A second round of QC performed at chromosome level includes GATK VQSR, removal of low complexity regions, removal of variants with excessive depth (for WGS only), removal of monomorphic variants, allele balance heterozygosity ratio filter, and hard filtering for SNPs and INDELs separately. After QC, all chromosomes are merged together for a final QC on SNPs and samples using PLINK1.9 following the same criteria described for the array-based genotyping data.

##### Data availability

WES is currently available for 1,069 participants, 578 of those classified as sporadic AD cases, and 382 as controls in the last clinical assessment (Table 5). Ethnically, most of them self-report as NHW, 1,021. There are 1,366,915 variants available with 98% call rate, 1,255,468 variants with MAF below 5%, and 1,186,359 variants with MAF below 1%. For WGS data, 2,074 participants’ sequences are available distributed as 928 sporadic AD cases, and 907 controls (Table 5). Similar to WES and the overall distribution of the Knight-ADRC participants, most of them identify as NHW, 1,807, followed by 240 AA. Variant wise, 85,891,726 high quality variants with a call rate greater than 98%, 79,568,847 variants with MAF below 5% and 75,450,819 with MAF below 1% are available.

#### In-house mutation screening

Autosomal Dominant AD (ADAD) is a rare form of AD characterized by the presence of specific missense mutations in the genes *APP, PSEN1*, or *PSEN2* with high penetrance and autosomal dominant inheritance^8^. At Washington University Knight-ADRC, there is one family with a mutation in *APP*, eleven families with mutations in *PSEN1*, and one family with a mutation in *PSEN2*. However, none of these families fully qualify for DIAN given their later age at onset and incomplete penetrance. In fact, it has already been reported by investigators at the Knight-ADRC that mutations in these three genes can also be found in individuals with later onset AD and without perfect penetrance^18^. There are other genetic variants that modify AD risk. On top of *APOE* ε4 allele, which is the major genetic risk factor^24^, *TREM2* variants are strongly associated with risk for developing AD^25^. There are 17 families with mutations in *TREM2* followed at the Knight-ADRC. Additionally, specific mutations in *MAPT*, *GRN*, and a repeat element in *C9or72f* are known to contribute to FTD^26^. There are three families with mutations in *MAPT*, five families with mutations in *GRN*, and three families with the *C9orf72*. In consequence, all samples from the Knight-ADRC participants undergo variant screening in the GHTO, and this data is included in the GHTO database.

#### Polygenic Risk Scores Calculation and availability

Polygenic risk scores (PRSs) allow calculation of background genetic risk of a given individual for a given trait assuming that a genome-wide association analysis (GWAS) is available. At the GHTO, PRS is calculated as part of the established pipeline prior to data release. The latest available GWAS summary statistics of several traits of interest are used to generate risk scores for all the individuals with the genotype data using PRSceV2.3^27^. Briefly, PRS are computed by calculating the sum of risk alleles weighted by the effect size estimate from the GWAS. Despite the AD-centric nature of the Knight-ADRC samples, the GHTO calculated PRS not only for AD, with and without the *APOE* locus, but also for Parkinson’s disease (PD)^28^, and Frontotemporal dementia (FTD)^29^, among others (Fig. 3c and Appendix 2). All PRSs are available for all samples with array-based genotyping data and have already been successfully leveraged to investigate the shared genetic structure between the earlier and later familial forms of AD, finding a high overlap among those forms^10^, and rate of progression^11^.

### Transcriptomic data

#### Overview of transcriptomic data: Bulk, single nuclei, spatial transcriptomics data, and tissue of origin

Similar to genomic data, transcriptomic technologies have evolved and become widely available with highly competitive prices. Unless somatic mutations are considered, transcriptomics is highly dependent on tissue of origin. Bulk transcriptomics was generated for a total of 630 brain samples from 490 unique participants. Parietal RNA-seq data is available for 487 individuals (total of 525 samples). The remaining 105 brain samples are distributed across frontal cortex (n = 40), temporal cortex (n = 26), and cerebellum (n = 39) brain regions and correspond to a total of 42 participants (Table 6).

**Table 6:** Transcriptomic data available at GHTO by brain region and library type (bulk or small).

	Bulk RNA-Seq	Small RNA-Seq
**Cerebellum**	39	
**Frontal Cortex**	40	
**Parietal**	525	330
**Temporal**	26	
**Total**	630	330

Single nuclei transcriptomic data is also currently available for 54 parietal brain samples. Brain spatial transcriptomics is available for a small proportion of samples (eight from temporal cortex, and one from parietal cortex). All the data described above contains coding and long non-coding transcriptome, however, in the context of the Knight-ADRC, there is also interest on the small non-coding transcriptome, thus, small transcriptome is also available on 330 knight-ADRC participants’ parietal brain samples.

Brain is the hallmark tissue affected by AD, however, blood is being intensively investigated as a source of biomarkers. Recently, PAXgene RNA tubes have been collected as part of each Knight-ADRC assessment. Of those, transcriptomic data is available for 2,334 assessments, corresponding to 1,485 individual participants. Additionally acellular transcriptomic data is available from 293 participants, and small transcriptomic for 164 Knight-ADRC participants (Table 5).

#### Bulk transcriptomic data

##### Sample preparation

RNA is extracted from 20 mg of brain, 2.5 mL of PAXgene preserved blood, or 500μL of plasma. For brain, frozen tissue is homogenized using metal beads immediately followed by cell lysis. Then, RNA is extracted in a Maxwell^®^ RSC 48 instrument using the Maxwell^®^ RSC simply RNA Tissue kit. For blood and plasma, there is no sample preprocessing, PAXgene mixed blood or plasma is directly loaded into the Maxwell^®^ RSC simply RNA Blood kit for purification or Maxwell^®^ RSC miRNA from plasma or serum kit respectively. Finally, brain and blood purified RNA are evaluated using RNA Screentapes on a 4200 TapeStation Instrument. Any RNA extraction not meeting quality standards (DV200 > 85%) is not processed for sequencing. For plasma, RNA is known to be degraded, thus only fluorometric quantification is performed, with a required concertation of at least 1.5 ng/μL. If additional tissue is available, a new extraction is conducted for those samples that fail QC.

Similar to the array-based genomic data, library preparation kits and sequencers have change and been updated over time. There are three batches of brain transcriptomic data; the first one contains 132 parietal brain samples that were spiked-inn with ERCC RNA ExFold Spike-Ins prior to ribodepletion (Ribo-Zero Gold kit) and library construction with TruSeq Stranded Total RNA Sample Prep kit. Finally, 100 million 150 bp pair ended reads per sample were targeted using a HiSeq 4000 (Illumina) instrument. The second batch contained 312 parietal brain samples from 288 unique participants that were ribo-depleted (kit) prior to library preparation using the Tru-Seq Stranded libraries with ERCC ExFold Mix. 70 million 150 bp pair ended reads per sample were targeted in an Illumina NovaSeq 6000 using S4 flowcells. The most recent data generation is composed by 184 brain samples from 89 participants. In here, Ribosomal RNA depletion was performed using FastSelect libraries. As in the prior batch, about 70 million 150 bp paired end reads were targeted in an Illumina NovaSeq 6000.

The blood transcriptomic data corresponding to 2,334 PAXgene preserved blood samples was generated at the same time. Both ribosomal RNA and globin were blocked using FastSelect, followed by library preparation (QIAGEN) and sequencing an average of 60 million 150 bp pair ended reads using an Illumina NovaSeq 6000. Finally, acellular plasma RNA was ribo- and globin-depleted prior to library preparation for Illumina sequencing using 1 ng of RNA as input. Due to the low input, libraries were cleaned from adaptor content prior to sequencing. Sequencing was performed in two batches, on the first one (91 plasma samples), 15 million 100 bp single-end reads were targeted using an Illumina HiSeq 2500, on the second one (245 plasma samples), the number of targeted reads increased to 40 million, and an Illumina NovaSeq 600 was used to generate them^30^.

##### Data processing and quality control

To obtain the linear counts, the GHTO teams follows standard pipelines. In summary, the initial quality of the data is firstly evaluated using fastqc; any sample with less than 1,000 reads or only library adaptor reads is removed at this stage. The remaining samples are aligned to the reference genome GRCh38 using STAR^31^ followed by generation of transcript counts using Salmon^32^. Alignment quality is evaluated using metrics collected with Picard: CollectRNAseqMetrics, MarkDuplicates, and CollectAlignmentSummaryMetrics (http://broadinstitute.github.io/picard). Additionally, transcript integrity numbers (TIN) are also calculated with the RSeQC^33^ package. All QC data is aggregated with multiqc^34^ for visual inspection. Any sample with multiple fastqc category failures, low percentage of mapped reads from STAR or Salmon (less than 50%, more than 20% of ribosomal RNA content, or low median TIN values) will be considered of poor quality and thus, removed from further analyses. Transcriptomic principal component is also calculated using the normalized counts from DESeq 2^35^ (using the *vst* function for brain and blood, and the *rlog* in plasma). Samples outside two standard deviations from the first two principal components are considered outliers. The complete pipeline for brain and blood transcriptomics can be found here: https://github.com/NeuroGenomicsAndInformatics/RNAseq_pipeline. The pipeline for plasma RNA-seq can be found here: https://github.com/Ibanez-Lab/PlasmaCellFreeRNA-AlzhiemerDisease.

Bulk transcriptomic data (from brain and blood only) is also processed to obtain circular transcript counts. Unlike traditional linear RNAs (which have 5′ and 3′ ends), circular RNA (circRNA) has a closed-loop structure that is unaffected by RNA exonucleases. Thus, circRNA has sustained expression and is less sensitive to degradation. Previous studies from Knight-ADRC investigators identified more than 100 circRNAs differentially accumulated in AD brains compared to controls, that are independent of changes in the linear RNA forms regardless of estimated brain cell-type proportions^12,20^. Given the importance of circRNA, the GHTO also provides circRNA quantification for those samples with enough sequencing depth (about 40 million reads). To obtain those counts, the original fastq or bam files are aligned to the human reference genome (GRCh38) using STAR^31^ in chimeric alignment mode. Other parameters are set according to the instruction manual for Detection of Circular RNA from Chimeric read (DCC)^36^. The collection of chimeric bam files is used as input for DCC that will quantify and collapse circular transcripts by the host gene.

##### Data availability

Overall, linear and circular transcriptome is available in brain for 371 AD cases, 7 ADAD cases, 37 controls, 14 DLB cases, 23 FTD cases, and 38 ADRD, and in blood for 312 AD cases, 2 ADAD cases, 801 controls, 2 DLB, 9 FTD, and 359 ADRD (Table 5). Linear acellular transcriptome is available for 333 plasma samples corresponding to 293 individual participants (134 AD Cases, 110 Controls, 16 DLB, 12 FTD, and 21 ADRD cases - Table 5).

#### Bulk small transcriptomic data

##### Sample preparation

The amount of purified RNA obtained using the protocols described above is enough to generate transcriptomic and small transcriptomic data. Library preparation is performed by RealSeq Biosciences (CA) using the RealSeq®-AC sRNA kit version 2. This is a new sRNA library preparation technology that reduces sequencing bias compared to previously used methods^37,38^. Briefly, previous methods use two adaptors, while this new technology leverages a single adaptor and a circularization reaction to reduce the sRNA sequence bias.

##### Data processing and quality control

Upon sequencing file receipt, libraries undergo adaptor trimming using cutadapt^39^, followed by alignment using bowtie2^40^, to available sRNA databases. The small transcriptome is populated by several families of sRNA, with private databases that the GHTO team leverages to obtain the counts. MicroRNA (miRNA) counts are obtained by aligning the trimmed sequences to miRbase^41^. Similarly, PIWI interacting RNAs (piRNAs) counts are obtained by aligning to piRBase^42^. GtRNAdb^43^ contains information about transfer RNA (tRNA), snoDB^44^ about small nucleolar RNAs (snoRNAs), and Rfam^45^ about vault RNAs (vtRNAs) and Y-RNAs. Finally, to obtain the count of small nuclear RNAs (snRNA), trimmed sequences are aligned to the human genome (GRCh38). Only perfectly aligned reads are quantified and QCed (each class of sRNAs is treated independently) using custom bash and R scripts (GitHub page under development: https://github.com/Ibanez-Lab/).

##### Data availability

Currently a total of 517 small RNA (sRNA) libraries, of which 330 samples (unique 330 individuals) are from brain (283 AD, 4 ADAD, 34 CO, 3 DLB, 3 FTD, 3 ADRD) and 187 are from plasma (from unique 164 individuals) (94 AD, 29 CO, 13 DLB, 11 FTD, 17 ADRD), have been generated from Knight-ADRC participants (Table 5).

#### Single nuclei RNA-seq

##### Sample preparation

Single nuclei isolation was carried out in parietal brain samples collected from 54 Knight-ADRC participants using a previously reported protocol (Neurogenomics And Informatics (protocols.io)). Briefly, a total of 200 mg of brain tissue were manually homogenized using a Dounce homogenizer and nuclei were isolated using a density gradient. Approximately 10,000 nuclei per sample and 50,000 reads per nuclei were target using the 10X Chromium single cell Reagent Kit v3^46^.

##### Data processing and quality control

Alignment and gene expression quantification was obtained using CellRanger (10X Genomics) following the directions from 10X Genomics pipelines. GRCH38 was used as reference genome. Quality control was performed in each sample individually using the Seurat package. Then, raw gene expression per sample were plotted to establish the inflexion points form the barcode-rank distribution that allowed to set threshold to exclude non-uniform regions of that distribution. Nuclei with high mitochondria were removed^47^ along with transcripts nor expressed in at least 10 nuclei. Doubles were removed using DoubletFinder^46,47^. One sample was removed due to low counts.

##### Data availability

Currently 53 brain samples are available corresponding to 9 healthy control brains, 31 sporadic AD, three presymptomatic AD, and eight corresponding to other dementia forms or mixed pathologies.

#### Spatial transcriptomics

##### Sample preparation

Fresh-frozen embedded brain tissue blocks from eight Kinght-ADRC participants were cryo-sectioned into 8–10 µm thick slices at optimal cutting temperature. Tissue slices were fixed following the fixation protocol provided by Vizgen and shipped to Vizgen. A panel of 260 genes were selected focusing on microglia and neurodegenerative disease related genes and pathways. Vizgen performed spatially resolved, single cell transcriptomic profiling measurements for the defined panel using Multiplexed Error-Robust Fluorescence *in Situ* Hybridization (“MERFISH”). Immunostaining for amyloid β (Purified anti-β-Amyloid, 17–24 Antibody, 4G8 clone, Biolegend), Phospho-Tau (Ser202, Thr205) Monoclonal Antibody (AT8) (ThermoFisher Scientific), and TDP-43 Polyclonal antibody (Proteintech) were performed on the same slides to obtain pathology information. DAPI and polyT staining were performed on the same slides to provide information for better cell segmentation.

##### Data processing and quality control

Vizgen performed cell segmentation using the CellPose algorithm implemented in the Vizgen Post-processing Tool (VPT) to draw cell boundaries and generates single-cell transcript expression level quantification matrix files. The same company performed initial data quality evaluation. Gene expression measured by MERSCOPE were highly correlated among processed samples (r = 0.90–0.98). In addition, the MERSCOPE measurements were highly correlations with a bulk brain cortex tissue RNA-seq data (r = 0.74–0.80). These results suggest that MERFISH measurement is highly reproducible and therefore is reliable for downstream data analysis. Then, single-cell genomics tool Seurat was used to perform initial data analyze. Cells with less than ten transcripts, or transcripts present in less than five cells were removed from analyses. An average of 105,320 (7,3725–136,388) cells were successfully measured with a median 47–194 (range 11–4344) transcripts, and a median number of 30–90 (range 10–235) genes detected. The immunostaining of protein TDP-43 did not produce expected detectable signal and the three samples were excluded from the analysis.

##### Data availability

Spatial transcriptomic data has been generated in a small subset of brain samples. Data is available from eight superior temporal gyrus, and one from the parietal region, corresponding to two control participants and seven AD cases (Table 5).

### High throughput Proteomics, Metabolomics, and Lipidomics

#### Overview of proteomic, metabolomic, and lipidomic data

High throughput proteomic, metabolomic and lipidomic datasets are becoming more common across cohorts. To date, there are several technologies available, at the Knight-ADRC, the technologies of choice have been Somalogic for proteomic data, and Metabolon for metabolomic, and lipidomic data. Proteomic, metabolomic, and lipidomic data is available in 514 parietal brain homogenates (404 unique participants), 1,079 CSF samples (933 unique participants), and 3,574 plasma samples (3,110 unique participants - Table 5, Fig. 2a,c,e).

#### High throughput proteomics

##### Sample preparation

Samples were collected following the protocols used at the Knight-ADRC^14,48^. Briefly, approximately 50 mg of fresh frozen brain samples corresponding to the parietal lobe were homogenized using metallic beads as explained above, and protein extract were performed as reported previously^14,48–50^ prior to submission to Somalogic, Inc. CSF samples were collected the morning after an overnight fast and were processed and stored at −80 °C until use. Unfasted plasma samples were collected at the time of clinical visit and immediately centrifuged and stored at −80 °C. No preprocessing was needed for CSF and plasma samples, prior to shipping.

##### Data processing and quality control

Somalogic, Inc performed the measurements and the initial data normalization to (i) control for inter-plate variances using the hybridization controls for intra-plate differences and median signals, and to (ii) and control for biological variance using an external reference. Then the GHTO team performed in depth QC, deeply described elsewhere^49,51–53^. In short, scale factors are computed for each aptamer in each plate. If the scale factor difference between any pairwise plate comparison is greater than 0.5, the aptamer measurement is considered unreliable and removed from further analysis. Then, a similar process is followed computing the coefficient of variation by aptamer and plate and performing all pairwise plate comparisons. If the difference is greater than 0.15 in any case, the aptamer is considered low quality and removed. With the remaining aptamers, outlier measurements are identified based on inter-quartile range (IQR) thresholds. Finally, two consecutive missing data thresholds (65% and then 85%) for aptamer with missing data and samples with missing aptamer values are used to are removed (Appendix 3 and 4).

##### Data availability

Proteomic data (Table 5) for brain homogenates (n = 412 | 320 AD cases, 6 ADAD cases, 27 controls, 9 DLB, 18 FTD, and 32 ADRD) was generated using the SomaLogic SOMAscan1.3k that provides 1,305 aptamers, of which 1,300 were retained after QC. Regarding plasma (n = 3,137 | 1266 AD cases, 10 ADAD cases, 1432 controls, 35 DLB, 45 FTD, and 349 ADRD) and CSF (n = 1064 |295 AD cases, 4 ADAD cases, 642 controls, 4 DLB, 10 FTD, and 109 ADRD) samples, proteomic data was generated using SOMAscan7k platform which measures the abundance of 7,584 aptamers^54^, with a retention of 6,905 aptamers for plasma, and 7,006 in CSF after QC. For some participants, more than one time-point measurement (plasma and CSF) or brain region are also available (not accounted for in Table 5 and Fig. 2). Olink HT1 data for 1,064 CSF samples has been recently generated and will be available soon.

#### High throughput metabolomic and lipidomic data

##### Sample preparation

Samples are prepared as described in the proteomic data.

##### Data processing and quality control

Similar to the proteomic data, Metabolon, Inc performed the preliminary QC on the metabolomic and proteomic data and provided data normalized by processing batch and volume. Additional QC was then conducted by the GHTO team as described elsewhere^55^. The nature of metabolomic and lipidomic data was considered during QC pipeline design. For example, non-xenobiotics (metabolites and lipids innate to the human body) are expected to be present in many samples, while xenobiotics (those that are foreign to the human system) can be largely missing due to their foreign nature. Thus, a first QC round assess overall missingness, and removes samples with more than 50% of metabolomic and lipidomic measurements missing. Then non-xenobiotics analytes missingness is computed; any analyte with more than 80% missingness across samples is removed. Missing values for the remaining non-xenobiotics are imputed as the minimum value observed for the given analyte^55^. Xenobiotics missingness is not assesses in this step, nor any imputation performed. Then, all values are normalized using log_10_ transformation and those with IQR of zero or low variance (less than 0.001) are considered non-informative and removed. Finally, IQR is used to remove outlier measurements, standard deviation of the two first metabolomic and lipidomic principal components is used to remove outlier samples (Appendix 5 and 6).

##### Data availability

Metabolomic and lipidomic data from brain, CSF, and plasma was generated via HD4 Metabolon’s untargeted Precision Metabolomics™ LC-MS (liquid chromatography–mass spectrometry) platform. High quality data is available in 441 brain homogenates (342 AD cases, 7 ADAD cases, 29 controls, 11 DLB, 19 FTD and 33 ADRD), with 797 analytes passing QC, 3,169 plasma samples (1285 AD cases, 9 ADAD cases, 1431 controls, 37 DLB, 48 FTD and 359 ADRD) with 1,508 metabolites and lipids passing QC, and 948 CSF samples (286 AD cases, 4 ADAD cases, 575 controls, 4 DLB, 10 FTD and 69 ADRD) with 456 analytes remaining after QC (Table 5). For some participants, more than one time-point measurement (plasma and CSF) or brain region are also available (not accounted for in Table 5 and Fig. 2).

### Epigenomic data

#### Overview

AD etiology is complex and not specific to a single genetic factor, or the dysregulation of one protein or transcript. Epigenetic changes could help explain the missing heritability not captured by genomic data and help determine functional variants in genome-wide significant loci that might led to transcriptomic, proteomic, or metabolomic changes. We have generated DNA methylation data from 444 parietal brain samples and 20 samples from whole blood.

#### Sample preparation

DNA is obtained from brain samples (50 mg) or whole blood (8 mL) using standard methods as described in the genomics section.

#### Data processing and quality control

The raw Illumina EPIC data is processed using the ENmix package^56^ followed by stringent quality control. In brief, the GHTO pipeline contains the following steps: Our stringent QC pipeline included the following steps: (i) calculation of the bisulphite conversion statistic and exclusion of samples that fail three or more control metrics; (ii) multidimensional scaling of probes on the X and Y chromosomes to confirm concordance between reported sex and genetic sex, with exclusion of mismatches; (iii) exclusion of poorly performing samples; (iv) removal of samples with more than 1% of probes with significant detection p-value (p > 0.05); (v) exclusion of samples with methylome principal component outside 2 standard deviation from the first three principal components; (vi) dye bias correction using the RELIC function in ENmix^57^; (vii) quantile normalization; (viii) exclusion of cross-hybridizing and SNP-related probes. Finally, methylation levels (beta values, β) at a given CpG site is calculated from the ratio of the methylated probe intensity to sum of methylated and unmethylated probe intensities. Finally, the ComBat function from the sva package is used to evaluate and remove the potential effects of technical variables (sample plate, array, and slide)^58^.

#### Data availability

Methylomic data was generated using the Illumina Infinium MethylationEPIC array that interrogates over 850,000 CpG and non-CpG sites, open chromatin, enhancers, DNase hypersensitive sites, and promoters. Parietal cortex brain methylation is available for 444 unique individuals (344 AD cases, 7 ADAD cases, 29 controls, 11 DLB, 19 FTD, and 34 ADRD) and 20 samples from whole blood (15 AD cases, and 5 controls – Table 5).

## Data Records

All data generated from Knight-ADRC participants have been and will be deposited at NIAGADS under the Knight ADRC collection (https://www.niagads.org/knight-adrc-collection). Data is only shared for participants with the appropriate consent level and always properly deidentified to ensure the anonymity of the participants. All the data is currently available at NIAGADS can be found under unique accession numbers distributed as follow: array-based genotyping data (NG00127), sequence data (NG00067), transcriptomic (NG00083), plasma acellular transcriptomics (NG00142), small RNA transcriptomics (NG00167 and NG00168), proteomic (NG00102; NG00128), metabolomic and lipidomic (NG00113), methylation (NG00114), single nuclei brain transcriptomic data (NG00108). VCF files corresponding to whole genome data can be found as part of the ADSP releases (ADSP-R2 for WES and ADSP-R4) along with the VCF files corresponding to whole exome data. Description of each dataset and the files included can be found in Table 7.

**Table 7:** NIAGADS Datasets Description.

Accession Number	Data Type	Data Files	Supporting Meta Data
NG00067	Whole Genome/Exome	NG00067.v12.txt: manifest all samples included on the release NG00067.v12.pdf: Release notes describing all files *.bam: bam files for all sequence samples	
NG00083	Bulk brain and blood Transcriptomic	Readme.txt: brief description of the files and the study Summaty_statistics_data.zip: summary statistics *.txt: flles with RNA lineal gene counts for each brain region *.txt: flles with RNA circular gene counts for each brain region	
NG00102	Brain, Plasma, and CSF Proteomics	NG00102.v1.txt: file describing all the files included on the dataset (28 files)	
NG00108	Single nuclei transcriptomics	NG00108.v2. txt: file describing all the files included on the dataset (60 files)	
NG00113	Brain, Plasma, and CSF Metabolomic	NG00113.v1.txt: file describing all the files included on the dataset (7 files)	
NG00114	Brain Methylomics	NG00114.v1.txt: file describing all the files included on the dataset (4 files)	
NG00142^30^	Plasma Acellular Transcriptomics	File name: discovery.data.txt, replication.data.txt, continuum.data.cdr05a.data.txt, continuum.data.cdr05b.data.txt, continuum.data.cdr1a.data.txt, continuum.data.cdr1b.data.txt, specificity.data1a.txt, specificity.data1b.txt Description: Each subset of individuals has a single txt file containing deidentified ID, age at draw, and all the columns corresponding to the normalize transcript counts at gene level.	File Name: pheno_submission_final.txt Description: Deidentified ID, age at draw, sex, APOE genotype, CSD, time in the freezer, biomarker levels and dementia diagnosis.
NG00127	Array-Based Genomic	NG00127.v1.txt: file describing all the files included on the dataset (10 files)	
NG00130	Brain, Plasma, CSF Proteomics	NG00130.v1.txt: file describing all the files included on the dataset (1462 files)	
NG00167	Bulk Brain Small Transcriptomics	File Name: deidentifiedID.fastq.gz Description: Fastq file for each of the included individuals.	File Name: pheno.txt Description: Deidentified ID, age at death, sex, diagnostic, sequencing pool.
NG00168	Plasma Acellular Small Transcriptomics	File Name: deidentified.fastq.gz Description: Fastq file for each of the included individuals.	File Name: pheno.txt Description: Deidentified ID, age at draw, sex, dementia diagnosis, sequencing pool.

### Browsers & data sharing

To enable other interested researchers to navigate the findings from several of our papers based on Knight ADRC data, we created and regularly update multiple web browsers:

#### The Online Neurodegenerative Trait Integrative Multi-Omics Explorer (ONTIME)

ONTIME web browser (https://ontime.wustl.edu) allows navigation of all the QTL data that currently includes more than 26,000 molecular phenotypes: proteomics and metabolomics in brain, CSF and plasma from European and African Americans^14^. It was built using PheWeb version 1.3.16^59^, an open-source tool for visualizing and sharing GWAS and PheWAS results. One of the key features of ONTIME is its interactive plot, which displays QTL data and allows users to explore the data in detail. ONTIME provides intuitive visual summaries at three levels of detail: (i) genome-wide summaries with traits with Manhattan and QQ plots, (ii) regional view using LocusZoom, and (iii) phenome-wide associations to highlight the association and p-value at the genetic variant across all proteins. All figures generated by ONTIME can be downloaded.

#### The multi-tissue proteomics browser

In addition to several QTL studies, we also performed differential abundance analysis to identify proteomic profiles not only for sporadic AD, but also for genetically defined AD subtypes (carriers for *TREM2* risk variants and autosomal dominant AD individuals) in Knight-ADRC data. We created a Shiny-based web portal (omics.wustl.edu/proteomics and https://proteomics.wustl.edu/csf/) to facilitate both exploration of our analysis and further investigation into individual proteins across disease status or sex. The browser provides three tabs. The first tab provides a brief description of data set and explanation. The second tab (Abundance Distribution) displays a table including proteomic abundance levels on each analyte that passed our QC process, along with its effect and p-value for each comparison presented here. The table allows the user to select a protein, which displays the distribution of the selected protein levels across disease status or sex. The third tab (Volcano Plots) displays the volcano plots for each comparison^52^.

## Technical Validation

**Fig. 4:**
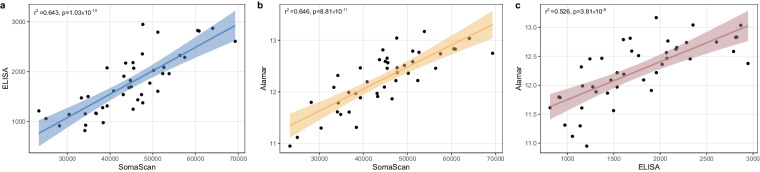
Two by two comparison of CSF soluble TREM2 levels measured using different technologies (**a**). CSF Soluble TREM2 levels correlation between high-throughput platform SomaScan and traditional ELISA (**b**). CSF soluble TREM2 measurement correlation between SomaScan and Alamar (NULISA) (**c**). Correlation between the measurements of soluble TREM2 in CSF using ELISA or Alamar technologies.

At the GHTO stringent practices and pipelines are in place to ensure the top quality of the released data. In this report, we have included all the technical and quality control thresholds, pipelines and parameters used during the processing of each data type. Briefly, and independently of the inclusion of multiple technical duplicates and biological replicas during data generation, the GHTO team:

- Calculate Principal Components: Genomic principal components are leveraged to adjust by genetic background (Fig. 3b). In general, HapMap or the Reference Genome are used as anchors. For quantitative traits such as transcriptomics or proteomics, those are used to identify outliers and batch effects.
- Identity By Descent (IBD): In the context of genomic data, we ensure that the technical and biological replicates are true replicas, and the known family structures match those of the samples. For technical replicas, unexpected matches help identify and solve potential errors during data generation. Additionally, samples with array-based genomic data and sequence based-data are also IBD cross-checked to confirm identity. In the context of transcriptomic data (excluding small RNAseq data), the RNA sequences are aligned, and variants are called to perform IBD. This ensures that the identity of the RNA samples (from brain, blood, or plasma) match those of DNA. Finally, methylome data also contains enough genotyped SNPs to confirm identity and validate technical replicates via IBD, and across platforms.
- Sex Check: Using genomic data, genomic sex is calculated with plink^21,60^ and compared to the reported sex. This not only ensures the quality of our phenotype-genotype matrices, but also helps (along with IBD) to identify and solve potential data generation errors. When genomic sex cannot be calculated using plink, chromosome balance from WES/WGS us used to determine genomic sex
- Proteomics validation: Large-scale proteomic assays are cost-effective platforms over traditional immunoassay-based methods, but their performance and thresholds need to be evaluated to supplement the already established immuno-assays for biomarker evaluation. We performed comparative analyses of SOMAscan and immunoassay-based protein measurements for five cerebrospinal fluid (CSF) proteins associated with AD and neurodegeneration (Fig. 4): NfL, Neurogranin, sTREM2, VILIP-1, and SNAP-25. Our data indicate that SOMAscan performs as well as immunoassay approaches for NfL, Neurogranin, VILIP-1, and sTREM2 indicating that SOMAscan can be an alternative to traditional immunoassay-based measures^49^.

## Usage Notes

AD is a complex, polygenic disease with genetic, cellular, pathologic, and clinical heterogeneity. More than 74 genetic loci associated with disease risk have been identified. However, the gene(s) driving these associations, their functional mechanisms, and how they influence disease development or progression have not yet been fully characterized. There is thus a need for novel, unbiased approaches to identify and describe the biological pathways that lead to AD, and to determine how these pathways interact with one another. An integrative biology approach combining clinical and molecular data will improve identification of disrupted pathways and networks that will help uncover novel, high-value therapeutic targets for the prevention and treatment of AD.

The multi-tissue multiomic data generated on the Knight-ADRC samples has already been used to identify novel genes, transcripts, and proteins implicated in disease, create new predictive models, and identify causal and potentially druggable targets. In the next section there are some examples of all the high-quality research happening at the GHTO. A compilation of all the papers published by GHTO investigators and collaborators can be found in Table 8. Overall, they represent very good examples of how curated high-throughput data, along with data integration will lead to a better understanding of AD pathobiology and to the identification of novel therapeutic approaches, and disease modifying targets.

**Table 8:** Compilation of the most relevant publications leveraging GHTO resources.

Fist Author	Title	Data Used
Western, D^51^	Proteogenomic analysis of human cerebrospinal fluid identifies neurologically relevant regulation and informs causal proteins for Alzheimer’s disease.	Array-Based Genomics CSF proteomics
Wang, C^55^	Unique genetic architecture of CSF and brain metabolites pinpoints the novel targets for the traits of human wellness.	Array-Based Genomics CSF & Brain Metabolomics
Ali, M^69^	Multi-cohort cerebrospinal fluid proteomics identifies robust molecular signatures for asymptomatic and symptomatic Alzheimer’s disease.	CSF Proteomics
Do, AN^70^	CSF proteomic profiling with amyloid/tau positivity identifies distinctive sex-different alteration of multiple proteins involved in Alzheimer’s disease.	CSF Proteomics
Wang, L^71^	Proteo-genomics of soluble TREM2 in cerebrospinal fluid provides novel insights and identifies novel modulators for Alzheimer’s disease.	Array-Based Genomics CSF proteomics
Cisterna-García, A^30^	Cell-free RNA signatures predict Alzheimer’s disease.	Plasma acellular Transcriptomics
Timsina, J^72^	Harmonization of CSF and imaging biomarkers in Alzheimer’s disease: Need and practical applications for genetics studies and preclinical classification.	CSF Proteomics
Brase, L^47^	Single-nucleus RNA-sequencing of autosomal dominant Alzheimer disease and risk variant carriers.	Brain Single Nuclei Transcriptomics
Oh, HS^73^	Organ aging signatures in the plasma proteome track health and disease.	Plasma Proteomics
Wang, D^74^	Frequency of Variants in Mendelian Alzheimer’s Disease Genes within the Alzheimer’s Disease Sequencing Project (ADSP).	Whole Genome Sequencing
Gorijala, P^75^	Alzheimer’s polygenic risk scores are associated with cognitive phenotypes in Down syndrome.	Array-Based Genomics PRS Calculations
Phillips, B^76^	Proteome wide association studies of LRRK2 variants identify novel causal and druggable proteins for Parkinson’s disease.	Array-Based Genomics CSF proteomics
Sung, YJ^48^	Proteomics of brain, CSF, and plasma identifies molecular signatures for distinguishing sporadic and genetic Alzheimer’s disease.	Brain, Plasma, & CSF Proteomics
Panyard, DJ^77^	Large-scale proteome and metabolome analysis of CSF implicates altered glucose and carbon metabolism and succinylcarnitine in Alzheimer’s disease.	Proteomics Metabolomics
Bradley, J^78^	Genetic architecture of plasma Alzheimer disease biomarkers.	Array-Based Genomics Plasma proteomics
Ali, M^79^	Large multi-ethnic genetic analyses of amyloid imaging identify new genes for Alzheimer disease.	Array-Based Genomics
Yang, C^80^	Mendelian randomization and genetic colocalization infer the effects of the multi-tissue proteome on 211 complex disease-related phenotypes.	Array-Based Genomics Brain & CSF proteomics
Holstege, H^81^	Exome sequencing identifies rare damaging variants in ATP8B4 and ABCA1 as risk factors for Alzheimer’s disease.	Whole Exome Sequencing
Li, F^82^	Weakly activated core neuroinflammation pathways were identified as a central signaling mechanism contributing to the chronic neurodegeneration in Alzheimer’s disease.	Brain Bulk Transcriptomics
Yang, C^14^	Genomic atlas of the proteome from brain, CSF and plasma prioritizes proteins implicated in neurological disorders.	Brain, Plasma, & CSF Proteomics
Dube, U^12^	An atlas of cortical circular RNA expression in Alzheimer disease brains demonstrates clinical and pathological associations.	Brain Bulk Transcriptomics
Li, Z^83^	The TMEM106B FTLD-protective variant, rs1990621, is also associated with increased neuronal proportion.	Brain Bulk Transcriptomics
Deming, Y^84^	The MS4A gene cluster is a key modulator of soluble TREM2 and Alzheimer’s disease risk.	Array-Based Genomics
Del-Aguila, JL^85^	TREM2 brain transcript-specific studies in AD and TREM2 mutation carriers.	Brain Bulk Transcriptomics
Fernandez, MV^16^	Evaluation of Gene-Based Family-Based Methods to Detect Novel Genes Associated With Familial Late Onset Alzheimer Disease.	Whole Genome Sequencing Whole Exome Sequencing
Cruchaga, C^10^	Polygenic risk score of sporadic late-onset Alzheimer’s disease reveals a shared architecture with the familial and early-onset forms.	Array-Based Genomics PRS Calculations
Fernandez, MV^18^	Analysis of neurodegenerative Mendelian genes in clinically diagnosed Alzheimer Disease.	Whole Genome Sequencing Whole Exome Sequencing

### Novel gene discovery

The genomic data generated by the GHTO has been included in the largest GWAS for AD, and FTD^29,61^, and several sequencing studies. Internally, GHTO investigators performed exome-sequencing in highly selected families, identifying and characterizing two novel genes for AD: *PLD3* and *UNC5C*^62,63^. These and subsequent studies paved the road for some of the largest sequencing studies for AD that are still ongoing such as the Alzheimer’s Disease Sequencing Project (ADSP; https://www.niagads.org/adsp/content/home) in which GHTO member hold leadership roles.

Most genetic studies for AD and other complex disorders rely on a case-control study design to identify risk variants. These approaches are not suitable to identify genetic variants that modify other aspects of the disease, eg. disease progression or age at onset. Using endophenotypes increase the power to identify those because they are less heterogeneous than clinical diagnoses and are more directly affected by genetic variation. The GHTO dataset is very rich on AD-endophenotypes, in consequence, multiple studies have integrated genomic information with quantitative endophenotypes to identify genes implicated on disease. There is special interest in CSF biomarkers as quantitative traits for genetic studies. GHTO investigators have performed the largest GWAS to date leveraging CSF amyloid beta, total tau, and phosphorylated tau and identified a total of seven loci (three novel). Later on, it was demonstrated that these variants were associated with other AD phenotypes, including risk for disease, rate of progression and tau pathology^15,64–66^. Further studies also demonstrated that *APOE* increases the risk of AD by affecting tau pathology independent of amyloid beta^67^, further supporting the use of endophenotypes.

### Circular RNAs play a role in Alzheimer disease pathogenesis

GHTO researchers demonstrated for the first time that brain circRNAs are causally associated with AD^12^. In AD, synapse lost is one of the events known to be implicated in the disease, interestingly, several studies support the hypothesis that circRNAs are enriched in the synapse. In consequence, the circRNA composition in the brains available from Knight-ADRC donors was assessed in a three-stage study design (discovery, replication and meta-analysis). There were 148 circRNAs significantly correlated with CDR after FDR correction, the top hits being circ*HOMER1* and circ*CDR1-AS*. The results were independent of the lineal forms of the host genes, as well as cell proportions, known to be a confounding factor in brain transcriptomic analyses. When building co-expression networks centered on the causal AD genes, several of the identified circRNA pertained to same pathways, supporting a causal role for circRNA in AD pathobiology. At the time, this was an important study in the field, as circRNAS were quantified and validated by real-time PCR for the first time in human brain samples and at genome-wide scale in large and well-characterized cohorts. It also demonstrated that circRNAs are likely to be implicated on complex traits, including AD. Thus, further research will help to understand the biological events that lead to disease.

### Multi-tissue proteomics help characterize disease mechanisms

GWAS for AD have identified more than 74 genetic regions associated with AD^61^. However, the gene driving the association for most of those regions remains unknow. In order to understand the pathological events that lead to disease we need to identify the genes driving those associations and investigate the underlying biology of those genes. To do that, GHTO researchers have generated and leveraged the high-throughput proteomic data in multiple neurological-relevant tissues that is available. The aim is to characterize the genetic architecture that governs protein levels and how these proteins modulate AD risk. To do so, protein quantitative trait loci (pQTLs) from CSF, brain and plasma were calculated. There were 292 pQTL loci (264 novel) for CSF, 181 (66 novel) for plasma and 73 (46 novel) for brain. Next, they investigated the causal effect of proteins levels in AD by employing Mendelian randomization approaches using study-wide significant pQTL as instrumental variables.

In late 2013, two independent studies reported that a heterozygous rare variant in *TREM2* p.R47H is significantly associated with AD risk, with an odds ratio similar to that of an individual carrying one APOE ε4 allele^68^. This finding was extremely important in the field as the effect size of *TREM2* was similar to that of *APOE*, the strongest known genetic risk factor for AD. TREM2 plays a critical role in microglial activation, survival, and apoptosis, as well as in AD pathogenesis. Researchers at the Knight-ADRC, previously reported the *MS4A* locus as a key modulator for soluble TREM2 (sTREM2) in CSF^15^. To identify additional novel genetic modifiers of sTREM2, they performed the largest GWAS and identified four loci for CSF sTREM2 levels in 3,350 individuals of European ancestry. This study provided novel insights into the *MS4A* and *TREM2* loci, two well-known AD risk genes, and identified *TGFBR2* and *NECTIN2* as additional modulators involved in TREM2 biology. It is important to note that the *MS4A locus is* also known to be associated with AD risk, but the mechanism by which it affects AD risk was unknown. This work had several important implications: 1) elucidates the functional mechanism by which *MS4A4A* affects AD risk, modulation of TREM2 levels; 2) demonstrates that TREM2 involvement in AD pathogenesis is not limited to mutation carriers; 3) pharmaceutical targeting of MS4A4A, TGFBR2 or NECTIN2 are valid therapeutic approaches to modulate TREM2 levels and in consequence, AD risk.

### Biomarker and prediction modeling

In order to identify pathological events leading to disease, GHTO researchers leveraged the brain, CSF and plasma high-throughput proteomic data and identified eight proteins in the brain, 40 in CSF, and nine in the blood associated with sporadic AD. In *TREM2* risk variant carriers, they identified and validated a unique proteomic profile that can distinguishing *TREM2* carriers from controls and sporadic AD cases. This study marked the first proteomic analysis of TREM2 variant carriers. These proteins were enriched in autophagy, amyloid formation, and response to cytokines and highlight that different processes lead to disease and specific prediction models can be generated depending on the genetic background of the person^48^.

Proteins are the natural and most exploited source of biomarkers. However, nucleic acids a minimally exploited source of biomarkers. Researchers at the GHTO have leveraged acellular plasma RNA molecules to build predictive models for preclinical AD^30^. They not only showed the potential of these biomarkers for early detection, but also demonstrated that they are specific to AD, independent of amyloid beta, and can potentially be used to follow-up disease progression.

### Supplementary information

Appendix 1

Appendix 2

Appendix 3

Appendix 4

Appendix 5

Appendix 6

## Data Availability

All the bioinformatic pipelines described in this manuscript can be found via Github at https://github.com/NeuroGenomicsAndInformatics, and https://github.com/Ibanez-Lab/. All laboratory protocols can be found via protocols.io at https://www.protocols.io/researchers/neurogenomics-and-informatics. No custom code was used to generate the data described in this manuscript.
