# Supplementary material for: Genetic and multi-omic resources for Alzheimer disease and related dementia from the Knight Alzheimer Disease Research Center
Source: Sci Data. 2024 Jul 12;11:768. doi: 10.1038/s41597-024-03485-9 (PMC11245521; doi:10.1038/s41597-024-03485-9)
Supplement: Supplementary file 1 — Appendix 1 [file 41597_2024_3485_MOESM1_ESM.docx]

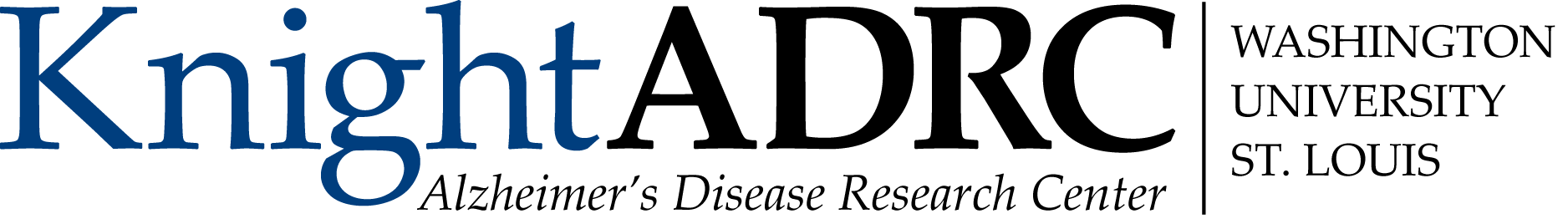


**Knight ADRC GWAS protocol**

Priyanka Gorijala^1,2^, Carlos Cruchaga^1,2,3^

1 Department of Psychiatry, Washington University School of Medicine, St. Louis, MO, USA

2 Neurogenomics and Informatics Center, Washington University School of Medicine, St. Louis, MO, USA

3 Hope Center for Neurologic Diseases, Washington University, St. Louis, MO, USA

Table of Contents

[Software requirements 2](file:///\\hydra.psych.wucon.wustl.edu\03-DryLab\02-Data\01-Array\03-AnalysisReady\02-GRCh38\03_Requests\202203_hg38\2022_10_MAP_Datafreeze\Knight-ADRC_GWAS_protocol_CC.docx#_Toc118892899)

[Introduction 3](file:///\\hydra.psych.wucon.wustl.edu\03-DryLab\02-Data\01-Array\03-AnalysisReady\02-GRCh38\03_Requests\202203_hg38\2022_10_MAP_Datafreeze\Knight-ADRC_GWAS_protocol_CC.docx#_Toc118892900)

[Pre-QC 3](file:///\\hydra.psych.wucon.wustl.edu\03-DryLab\02-Data\01-Array\03-AnalysisReady\02-GRCh38\03_Requests\202203_hg38\2022_10_MAP_Datafreeze\Knight-ADRC_GWAS_protocol_CC.docx#_Toc118892901)

[QC 4](file:///\\hydra.psych.wucon.wustl.edu\03-DryLab\02-Data\01-Array\03-AnalysisReady\02-GRCh38\03_Requests\202203_hg38\2022_10_MAP_Datafreeze\Knight-ADRC_GWAS_protocol_CC.docx#_Toc118892902)

[Sex check 4](file:///\\hydra.psych.wucon.wustl.edu\03-DryLab\02-Data\01-Array\03-AnalysisReady\02-GRCh38\03_Requests\202203_hg38\2022_10_MAP_Datafreeze\Knight-ADRC_GWAS_protocol_CC.docx#_Toc118892903)

[Relatedness check 5](file:///\\hydra.psych.wucon.wustl.edu\03-DryLab\02-Data\01-Array\03-AnalysisReady\02-GRCh38\03_Requests\202203_hg38\2022_10_MAP_Datafreeze\Knight-ADRC_GWAS_protocol_CC.docx#_Toc118892904)

[Pre-imputation 6](file:///\\hydra.psych.wucon.wustl.edu\03-DryLab\02-Data\01-Array\03-AnalysisReady\02-GRCh38\03_Requests\202203_hg38\2022_10_MAP_Datafreeze\Knight-ADRC_GWAS_protocol_CC.docx#_Toc118892905)

[Imputation 6](file:///\\hydra.psych.wucon.wustl.edu\03-DryLab\02-Data\01-Array\03-AnalysisReady\02-GRCh38\03_Requests\202203_hg38\2022_10_MAP_Datafreeze\Knight-ADRC_GWAS_protocol_CC.docx#_Toc118892906)

[Post-Imputation 7](file:///\\hydra.psych.wucon.wustl.edu\03-DryLab\02-Data\01-Array\03-AnalysisReady\02-GRCh38\03_Requests\202203_hg38\2022_10_MAP_Datafreeze\Knight-ADRC_GWAS_protocol_CC.docx#_Toc118892907)

[Merging arrays 8](file:///\\hydra.psych.wucon.wustl.edu\03-DryLab\02-Data\01-Array\03-AnalysisReady\02-GRCh38\03_Requests\202203_hg38\2022_10_MAP_Datafreeze\Knight-ADRC_GWAS_protocol_CC.docx#_Toc118892908)

# Software requirements

Software needed to implement the entire QC protocol

- PLINK version 1.9^1^
- R version 3.5.2 ( <https://www.r-project.org/> )
- Need the following R packages: dplyr ^2^, tidyr ^3^ , ggplot2^4^, spgs^5^, tidyverse^6^, ggtern^7^, data.table^8^
- VcfCooker ( <https://genome.sph.umich.edu/wiki/VcfCooker> )

# Introduction

- This protocol outlines the steps for conducting standard quality control (QC) for pre-imputation genotype data (common variants and rare variants) and standard practices for pre-imputation data preparation, imputation, and post-imputation data management.
- (*Note: The initial steps are applied to pre-imputation genotype datasets only and not imputed genotype data*.)
- Before submitting the genotype data for imputation, the data must undergo a few steps such as Pre-QC, QC, and pre-imputation.

# Pre-QC

- Input file formats: Standard PLINK-readable binary pedigree (BED/BIM/FAM) files are used as input to all the Pre-QC, QC, Pre, and post-imputation steps.
- Check if files can be read by PLINK (https://www.cog-genomics.org/plink) before moving to the next steps
- Before any processing confirm the array and genome build of the data and download the manifest file corresponding to the array build.
- In the Pre-QC perform some initial checks on the plink file for data evaluation such as the presence of phenotype data, sex code in the fam file, duplicates by sample ID name, and check for the presence of 0/missing/Indels in the allele info.
- Filter out the variants with both alleles missing information (plink does not allow to update such type of alleles).
- Update the Indels and one allele missing variants in the plink using the manifest file (Use the SNP column in the manifest to update allele info). Double-check for the presence of Indels/missing alleles after the update.
- Check for any variants lacking chromosome (Chr) and position information, and rectify by referencing either the manifest or dbSNP data to update accordingly.
- Identify duplicate variants using plink –list-dup-var. Filter the variants based on their call rate and call correlation (Call correlation threshold depends on the individual data distribution – to fix the Call correlation threshold, plot the distribution graph for correlation).
- We retain duplicate variants with different alleles (Multiallelics).
- Double check for the presence of duplicate variants with plink –list-dup-var.

# QC

- Investigate Missingness per SNP and individual.
- Two different thresholds are used to filter the data: a relaxed threshold of > 20% missing data in SNP and samples followed by a stringent threshold of >2% Missingness in SNP and samples
- If more than 1% of variants dropped, consider reviewing the genotype calling process
- Re-estimate call rate/Missingness rate.

## Sex check

- Update the sex info in the plink file (If sex info is not available in the fam file) using either demographics or clinical data file
- Prior to executing the sex check in Plink, utilize the "--split-x b38" command (b38 if data is on GRCH38 genome build, b37 if data is on GRCH37) to represent the X chromosomes pseudo-autosomal region as a separate 'XY' chromosome as preferred by Plink.
- Prune SNPs using Plink --indep-pairphase 20000 2000 0.5 followed by --Check-sex on pruned SNPs (this is recommended).
- Next flag the samples with discordance between phenotypic and genotypic sex. And investigate the discordance by digging into the phenotypic data.
- Extract autosomal variants from the plink file and estimate the *P*-value for Hardy-Weinberg Equilibrium (HWE) and filter out SNPs with *P*_HWE_<10^-6^
- Prune the autosomal HWE filtered file using --indep-pairwise 50 5 0.2
- Then perform Heterozygosity rate check on pruned SNPs and flag the samples deviating more than 3 standard deviations from the heterozygosity rate mean, but do not remove them as the heterozygosity mean deviation could be due to diversity in few populations and not a quality issue.

## Relatedness check

- Note: Using only genotype SNPs and not imputed SNPs is strongly recommended due to the variable quality of imputation and incompatibility of genotype probability data with relatedness check tools.
- Perform IBD check using the “--genome" command in PLINK, on autosomal variants that were pruned for LD and filtered for variant Missingness, HWE, and Minor allele frequency (--indep-pairwise 50 5 0.2, --geno 0.0001, --hwe 0.05, and --maf 0.15)
- Individuals who displayed excess relatedness were Identified and categorized as duplicates (pihat = 0.95 - 1) and first-degree relatives (pihat = 0.45-0.55). Pihat ranges differ with the datasets and were selected based on the data distribution of the pihat values.
- Keep 1 sample related to other samples if singletons, if$\hat{\pi}>0.95$. Keep the samples with the highest call rate
- Among related individuals, verify that the observed genetic relationship is consistent with the reported relationship, and flag if inconsistencies persist.
- Categorize the inconsistencies in the relationship into expected, unexpected, and expected missing duplicates.
  - When an expected match is found, add that to the list of expected duplicates maintained in the lab.
  - When an unexpected match was found try to determine if this is possible and the participant was recruited twice and provided a different ID
    - We check sex, DOB? Double-enrolled?
    - Ideally confirmed in the clinic by identical names? If determined that two samples come from the same individual, this is now an expected match.
  - If you cannot explain the match, then it means one of the samples is mislabeled. But we must identify which one is mislabeled.
    - Use family structure or sex mismatch data to determine which sample is correctly labeled, and which is incorrectly labeled.
    - If we are confident about which individual the samples came from; you can fix the incorrect one.
    - And if you cannot explain it, then BOTH individuals get labeled as unknown and removed. Must give the same unknown ID, since we know both samples came from the same individual. We do not know who that individual is. (In the future we might be able to figure it out – and then both samples will be fixed. We might even drop one sample in the future since we do not want to double-count.) They are both left in for now, since they have these unknown IDs and will never be used in any analysis. This data is never released.
  - When an expected missing duplicate is found, investigate if you lost those samples in the QC filtering, if not report those samples.
- After resolving IBD issues, in the next step combine Autosomal and non-autosomal variants to create the final QC’ed file
- Perform final check for duplicate variants by chr, pos, and alleles to take care of the same position warnings in the final merge step, filter variants as described in the Pre-QC.

# Pre-imputation

- The variants were lifted over to GRCH 38 build using Liftover chain lift19to38, no need for this step if the data is on the 38 build.
- In the LiftOver convert Chr 23,24,25,26 to X, Y, XY, and M and merge x and xy Chr’s.
- Create a bed file for Liftover - had to do the start position -1 because the bed is 0-based according to UCSC.
- After mapping the coordinates, exclude the variants in alternative or random chromosomes that failed LiftOver. Create separate text files with the chr and position info of the variants and use them to update the plink file to hg38.
- After LiftOver perform pre-imputation which includes:
  - Filtering mitochondrial variants and all InDels.
  - Variants’ strand, position, and reference allele assignment were updated using VcfCooker. Variants names were updated to chr:pos:ref:alt.
  - Update the variants strand, position, and reference allele assignment using VcfCooker.
  - Next update the variant name to a standard format chr:pos:ref:alt.
  - Finally split the file into separate chromosomes and sort based on the variant positions.
  - Only autosomal chromosomes were uploaded to the TOPMed server for imputation.

# Imputation

Imputation was performed using the NHLBI TOPMed Server. Standard instructions to use the server are as follows:

- NHLBI TopMed Server: <https://imputation.biodatacatalyst.nhlbi.nih.gov/>
- Register to use the server
- TOPMed imputation uses Minimac4 (based on MaCH)
- Once data is ready (Autosomal variants data from the pre-imputation step) select, or input the following options:
  - Enter Job name (optional)
  - Reference panel: ‘TOPMed r2’
  - Input Files: File Upload
  - Select all 22 chromosomes for simultaneous imputation
  - Array Build: GRCh38/hg38
  - Rsq filter: 0.3
  - Phasing: Eagle v2.4 (phased output)
  - Population: vs. TOPMed Panel
  - Mode: Quality Control & Imputation
  - AES 256 encryption not checked
  - Select - I will not attempt to re-identify or contact research participants.
  - Select - I will report any inadvertent data release, security breach or other data management incident of which I become aware.
  - And click ‘Submit job’
  - Wait for files to upload correctly (without reported error)
  - When the imputation is completed, a notification e-mail will be sent with a password and a link to download the imputation results.

# Post-Imputation

- TOPMED provides wget commands to download the data, follow that and download the data into the required folder
- Perform md5sum check after downloading TOPMED imputation results.
- Unzip the downloaded files using the password given by the TOPMED server and save the password for future use.
- Convert all 22 vcf files to plink format binary files (BED/BIM/FAM)
- Then merge all the imputed chromosomes (files in plink format)
- Estimate the P-value for Hardy-Weinberg Equilibrium (HWE) of the imputed data and filter out SNPs with *P*_HWE_<10^-6^
- In Parallel convert the pre-imputed vcf file (the file before splitting into individual chromosomes) into plink format
- Remove mismatch alleles from this file
- Now merge the HWE-filtered imputed file with invalid and mismatched allele-filtered unimputed data using merge-mode 2 in Plink.
- Identify duplicate variants using plink –list-dup-var. Filter the variants as described in the Pre-QC
- Temporarily remove chrY, and filter for SNP call rate of 98%, then add chrY back.
- Double-check the data for duplicate variants using –list-dup-var in Plink.
- Update the sex info in the Plink file
- If the initial data was received on two or multiple batches, follow the same steps from start to end on each batch separately and then merge the two or multiple batches using Plink default merge mode.
- Look for the duplicate variants after merging the batches. Filter out the following variants after merge:
- Same position-same allele and same position-inverted alleles (delete both in the pair- these are considered as errors from TOPMED imputation so remove both).
- Check for allele swaps (ref and alt allele swaps) at this stage and fix if any by setting the reference allele to a2 in Plink.
- Upon completing the post-imputation phase, apply diverse minor allele frequency (MAF) and genotype (geno) filters to confirm the high quality of the data.

# Merging arrays

- All the individual arrays after QC will be merged to prepare a data freeze for a year.
- Merge all the arrays processed one by one. This process helps in identifying where the issues are arising (if any) from.
- We look into the warnings given by Plink after each merge. We mostly end up with the same position warnings. Find out whether the same position warnings were the same alleles, flipped, or Multiallelics. Remerge after flipping the variants to see if it can resolve the same position warnings.
- Double check for the presence of duplicate variants with plink –list-dup-var.
- Quality of the data will be accessed after each merge by looking at the variants passed after multiple geno and maf filters as shown in **Table 1** (geno 0.02, maf 0.05; geno 0.02, maf 0.02; geno 0.02, maf 0.01; geno 0.05, maf 0.05; geno 0.05, maf 0.02; geno 0.05, maf 0.01; geno 0.1, maf 0.05; geno 0.1, maf 0.02; geno 0.1, maf 0.01). This will give an idea about how many variants passed QC after running all these filters and we can see whether they are in the range of common variants.
- If the variants passed the geno and maf filters were not in the expected range the data will undergo further quality control steps such as looking at the maf and call rate of the missing variants after running the above filters.
- SNP name was set to chr:pos:ref:alt during the liftover process for all the arrays and during the merge we checked for the allele discrepancy in variant name and a1/a2 alleles for all the arrays. If any discrepancies were found they will be addressed by assigning an alternate allele to a1 and reference allele to a2 in the plink file.
- After merging all the arrays we need to check for the IBD and sex mismatches and resolve the issues (if any) as mentioned above under the subheadings “Relatedness check” and “Sex check” respectively. After IBD and sex check samples with unresolved issues will be flagged with UNK (unknown) IDs in the plink file. Cross-array and cross-study duplicates will be identified during this step and will be noted cautiously.

|  |  |  |  | **98% call rate** | | |
| --- | --- | --- | --- | --- | --- | --- |
| **Year** | **Array** | **#Samples** | **#Variants** | **MAF 5%** | **MAF 2%** | **MAF 1%** |
| 201410 | UK BioBank | 69 | 13,764,334 | 6,369,031 | 8,062,434 | 9,238,626 |
| 201310 | Omni1-Quad | 66 | 16,092,402 | 6,170,602 | 7,732,446 | 8,771,706 |
| 201307 | OmniEx | 160 | 21,898,396 | 6,549,085 | 8,496,665 | 10,489,400 |
| 201112 | OmniEx | 276 | 29,909,687 | 6,388,114 | 8,189,465 | 10,204,969 |
| 201003 | 660W | 801 | 30,171,705 | 6,216,585 | 7,697,105 | 8,837,057 |
| 201412 | CoreEx | 407 | 32,792,423 | 6,339,403 | 7,993,011 | 9,653,156 |
| 201209 | OmniEx | 178 | 33,257,406 | 6,227,287 | 7,742,747 | 8,889,117 |
| 201509 | CoreEx | 422 | 39,544,393 | 6,317,233 | 8,064,462 | 9,978,610 |
| 201703 | NeuroX2 | 842 | 46,331,443 | 6,252,956 | 7,794,380 | 9,221,889 |
| 201812 | GSAv2 | 408 | 55,342,191 | 6,351,203 | 7,932,087 | 9,323,421 |
| 201711 | CoreEx | 114 | 59,181,991 | 6,227,144 | 7,817,626 | 9,400,866 |
| 201303 | OmniEx | 526 | 96,950,681 | 6,238,532 | 7,721,123 | 8,947,230 |
| 201506 | OmniExEx | 97 | 35,590,292 | 6,402,420 | 8,085,372 | 9,674,206 |
| 201603 | OmniExEx | 162 | 69,143,148 | 7,595,676 | 9,593,783 | 10,748,679 |
| 201612 | Human1M-Duov3 | 139 | 26,264,358 | 8,661,601 | 12,273,633 | 15,855,681 |
| 201802 | GSAv1 | 121 | 37,814,069 | 6,316,106 | 8,050,341 | 9,129,309 |
| 201903 | GSAv1 | 256 | 71,147,069 | 6,405,651 | 7,898,702 | 8,941,590 |
| 202103 | GSAv3 | 1,130 | 77,756,570 | 5,740,083 | 7,135,182 | 8,871,366 |

**Table 1** Details of all the Knight ADRC arrays with the sample numbers and variants. MAF 5%, 2% and 1% columns show the number of variants passed at 98% call rate and different minor allele frequency thresholds.

In summary all the samples were genotyped using Omni1-Quad, OmniEx, 660W, CoreEx, NeuroX2, GSA, and Human1M-Duov3 arrays. They were imputed by the TOPMed Imputation Server employing the TOPMed Reference Panel and variants with imputation quality of Rsq > 0.30 were retained. We applied standard quality control measures to each array separately before merging. In summary, single nucleotide variants (SNPs) and individuals were filtered for ≥ 98% call rate, and autosomal SNPs not in Hardy-Weinberg equilibrium (P-HWE <10-6) were filtered out. For the sex check, we split chr X to represent the X chr’s pseudo-autosomal region as a separate 'XY' chr and performed the sex check on pruned SNPs. Samples with discordance between phenotypic and genotypic sex were removed after this check. We also identified duplicates and familial relatedness by having identity-by-descent (IBD) estimates and selected only unrelated samples (IBD<0.25). All the quality control procedures were performed using Plink1.9 (<http://www.cog-genomics.org/plink2>).

We have 4843 participants and 160,506,717 variants that passed QC. Among those 4173 were Europeans, 626 Africans, 33 admixed Americans, and 11 Asians, determined by genetic principal components. At a 98% call rate 2,821,868 SNPs have MAF < 5%, 1,537,512 SNPs have MAF <2% and 662,110 SNPs have MAF <1% as shown in **Table 2**.

|  | | | **98% call rate (Variants with MAF<5%,2%,1%)** | | |
| --- | --- | --- | --- | --- | --- |
| **Cohort** | **#Samples** | **#Variants** | **MAF 5%** | **MAF 2%** | **MAF 1%** |
| Knight-ADRC | 4843 | 160,506,717 | 2,821,868 | 1,537,512 | 662,110 |

**Table 2** Details of all the Knight ADRC samples with the sample numbers and variants. MAF < 5%, 2%, and 1% columns show the number of variants passed at 98% call rate and different minor allele frequency thresholds

**References:**

1. Purcell S, Neale B, Todd-Brown K, et al. PLINK: A Tool Set for Whole-Genome Association and Population-Based Linkage Analyses. *The American Journal of Human Genetics*. 2007;81(3):559-575. doi:10.1086/519795

2. Wickham H, FR, HL, & MK. dplyr: A Grammar of Data Manipulation. R package version 1.0.7. Published 2021. https://CRAN.R-project.org/package=dplyr

3. Wickham H, & HL. tidyr: Tidy Messy Data. R package version 1.1.3. Published 2020. https://CRAN.R-project.org/package=tidyr

4. Wickham H. ggplot2: Elegant Graphics for Data Analysis. . Springer-Verlag New York.

5. Bivand R. spgs: Statistical Patterns in Genomic Sequences. R package version 1.7.2. Published 2019. https://CRAN.R-project.org/package=spgs

6. Wickham H, Averick M, Bryan J, et al. Welcome to the Tidyverse. *J Open Source Softw*. 2019;4(43):1686. doi:10.21105/joss.01686

7. Hamilton N (n. d. ). ggtern: An Extension to “ggplot2”, for the Creation of Ternary Diagrams. R package version 3.3.0. https://CRAN.R-project.org/package=ggtern

8. Dowle M, & SA. data.table: Extension of data.frame. R package version 1.14.2. Published online 2021. https://CRAN.R-project.org/package=data.table
