# Supplementary material for: Genetic and multi-omic resources for Alzheimer disease and related dementia from the Knight Alzheimer Disease Research Center
Source: Sci Data. 2024 Jul 12;11:768. doi: 10.1038/s41597-024-03485-9 (PMC11245521; doi:10.1038/s41597-024-03485-9)
Supplement: Supplementary file 2 — Appendix 2 [file 41597_2024_3485_MOESM2_ESM.docx]

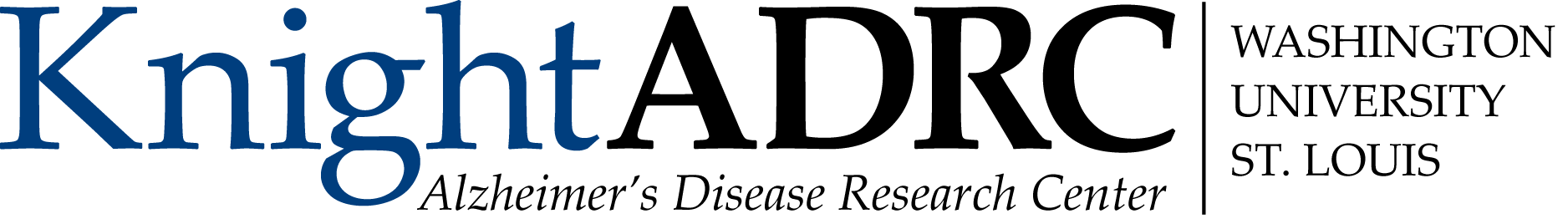


**Polygenic Risk Score Analysis**

Priyanka Gorijala^1,2^, Carlos Cruchaga^1,2,3^

1 Department of Psychiatry, Washington University School of Medicine, St. Louis, MO, USA

2 Neurogenomics and Informatics Center, Washington University School of Medicine, St. Louis, MO, USA

3 Hope Center for Neurologic Diseases, Washington University, St. Louis, MO, USA

**Introduction:**

Polygenic risk scores (PRSs^1^) are constructed by summing the risk alleles associated with a specific phenotype in each person, weighted by the effect sizes derived from comprehensive Genome-wide Association Studies (GWAS) related to that phenotype. With larger GWAS datasets and more significant findings, PRS becomes more robust, capable of elucidating a greater proportion of variance. This method provides a more precise estimation of individual risk compared to assessing genetic variations independently. PRS facilitates a deeper understanding of disease prognosis, enabling the development of early intervention strategies and refining criteria for participant selection in clinical trials. Additionally, PRS analysis can unveil the genetic interplay between co-existing complex traits^2^ by investigating the pleiotropic effects of markers identified in one trait on another^3^.

**Methods:**

For the calculation of Polygenic Risk Scores (PRS) for Alzheimer’s disease (AD)^4^, Parkinson’s disease (PD)^5,6^, and Frontotemporal dementia (FTD)^7^, we utilized the most recent Genome-wide Association Studies (GWAS) summary statistics specific to each condition. PRS were computed for all individuals with available genotype data (N = 4843) using PrsiceV2.3. This tool employs a clumping and thresholding approach, wherein variants in linkage disequilibrium are removed, and only those strongly associated with the trait are retained^1^. By applying a P-value threshold (e.g., p < 5 x 10^-8^), PrsiceV2.3 ensures that only highly significant variants contribute to the PRS calculation, effectively reducing the influence of less relevant variants to zero. PRS were calculated at a genome-wide threshold of 5 x 10^-8^ for AD and PD risk, while for FTD risk, the next best threshold of 5 x 10^-5^ was used due to the absence of remaining SNPs for risk assessment after clumping and thresholding at the genome-wide level. In the case of AD risk PRS, scores were generated both including and excluding the *APOE* region to better comprehend risk factors beyond *APOE* (GRCH38: 19:43907927 to 19:45908810). Finally, PRS were standardized using the population mean and standard deviation. PRS calculation is as follows:

**PRS_j_ = ∑_i_ (S_i_×G_ij_) – Mean (PRS) / SD(PRS)**

Where S is the summary statistic of the i^th^ effect allele

And G is the number of effect alleles observed for j^th^ individual.


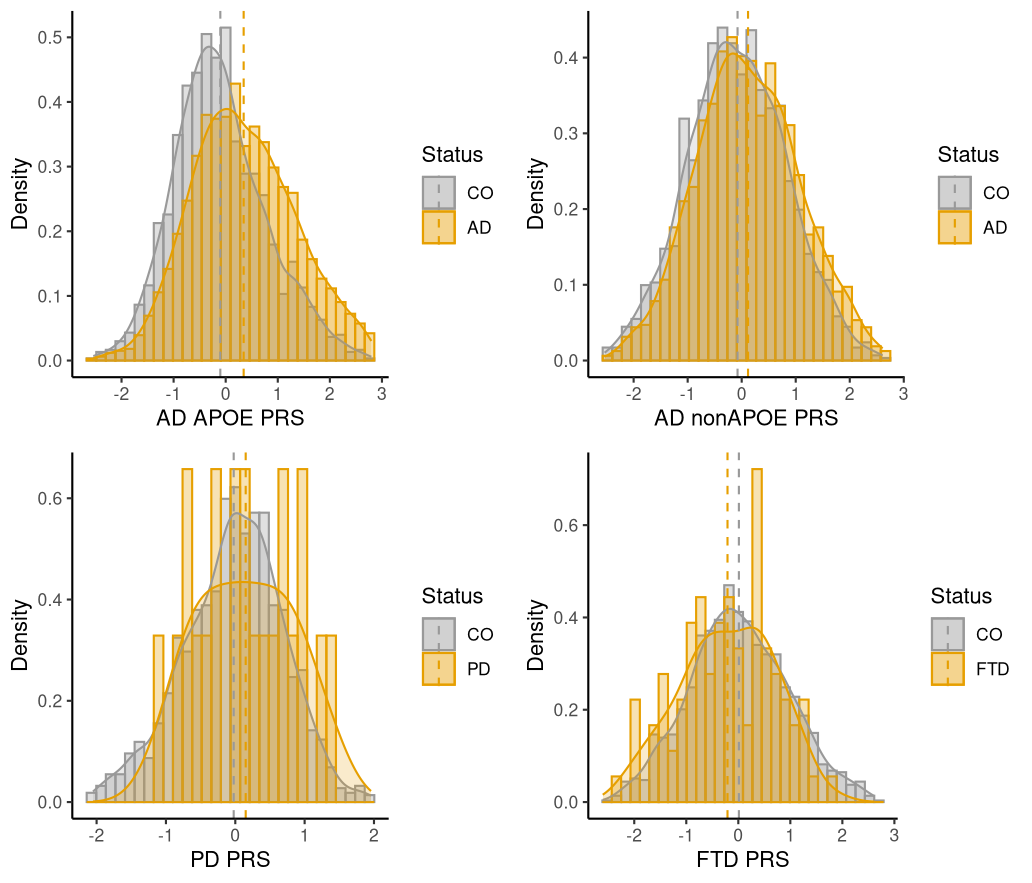


**Figure 1**: Distribution of PRS stratified by case control status at genome-wide P value threshold for A) AD risk PRS with APOE, B) Without APOE, C) PD risk PRS, and D) FTD PRS at 5 x10^-5^ threshold.


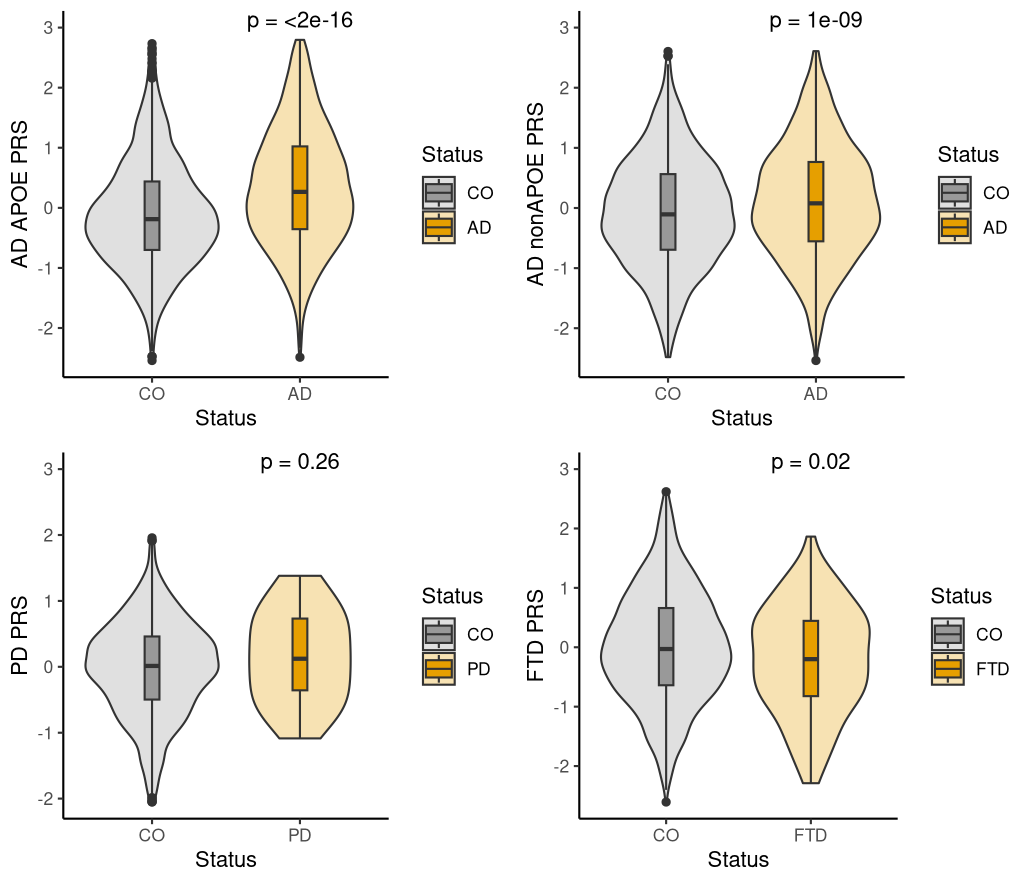


**Figure 2**: Violin plots visualizing the differences in PRS stratified by case-control status at genome-wide threshold for A) AD risk PRS with APOE, B) Without APOE, C) PD risk PRS, and D) FTD PRS at 5 x10^-5^ threshold.

| **Status** | **No of Samples** | **AD** ***APOE* PRS** | **AD non*APOE* PRS** | **PD PRS** | **FTD PRS** |
| --- | --- | --- | --- | --- | --- |
| AD | 1828 | 0.388 | 0.157 | -0.051 | 0.062 |
| ADAD | 7 | 1.073 | 0.426 | -0.236 | 0.736 |
| ALS | 1 | -0.618 | 0.023 | 0.941 | 0.545 |
| PD | 24 | -0.056 | 0.008 | 0.151 | 0.223 |
| DLB | 81 | 0.243 | 0.094 | -0.023 | 0.068 |
| FTD | 101 | 0.014 | 0.065 | -0.046 | -0.234 |
| CO | 1648 | -0.085 | -0.078 | -0.079 | 0.016 |
| OT | 797 | 0.113 | 0.136 | 0.001 | -0.003 |
| NA | 356 | 0.061 | 0.032 | -0.011 | -0.026 |
| **Table 1**: A summary of the mean polygenic risk scores at genome-wide threshold for all four traits AD with *APOE*, AD without *APOE*, PD, and FTD for each of the case-control status categories. Abbreviations: AD - Alzheimer’s disease, ADAD - Autosomal Dominant Alzheimer’s disease, ALS – Amyotrophic lateral sclerosis, PD- Parkinson’s disease, DLB – Dementia with Lewy bodies, FTD – Fronto temporal dementia, CO – Controls, OT – Others, NA- Status not available, PRS – Polygenic risk score, *APOE* – Apolipoprotein. | | | | | |

| **Variable** | **Odds Ratio** | **2.50% CI** | **97.50% CI** | **P value** | **N cases** | **N controls** |
| --- | --- | --- | --- | --- | --- | --- |
| AD *APOE* PRS | 3.08 | 2.60 | 3.65 | 1.10E-38 | 1828 | 1648 |
| AD non*APOE* PRS | 1.60 | 1.36 | 1.88 | 2.04E-08 | 1828 | 1648 |
| PD PRS | 1.08 | 0.43 | 2.74 | 8.69E-01 | 24 | 1648 |
| FTD PRS | 0.71 | 0.43 | 1.16 | 1.74E-01 | 101 | 1648 |
| **Table 2**: Association results of extreme tertiles of the PRS at genome-wide threshold derived for each of the AD, PD and FTD categories and compared to controls. Abbreviations: N – Number of samples in each category, PRS – Polygenic risk score, APOE – Apolipoprotein. AD - Alzheimer’s disease, PD- Parkinson’s disease, FTD – Fronto temporal dementia. | | | | | | |

References:

1. Choi SW, Mak TSH, O’Reilly PF. Tutorial: a guide to performing polygenic risk score analyses. *Nat Protoc*. 2020;15(9):2759-2772. doi:10.1038/s41596-020-0353-1

2. Cruchaga C, Del‐Aguila JL, Saef B, et al. Polygenic risk score of sporadic late‐onset Alzheimer’s disease reveals a shared architecture with the familial and early‐onset forms. *Alzheimer’s & Dementia*. 2018;14(2):205-214. doi:10.1016/j.jalz.2017.08.013

3. Clarke TK, Hall LS, Fernandez-Pujals AM, et al. Major depressive disorder and current psychological distress moderate the effect of polygenic risk for obesity on body mass index. *Transl Psychiatry*. 2015;5(6):e592-e592. doi:10.1038/tp.2015.83

4. Bellenguez C, Küçükali F, Jansen IE, et al. New insights into the genetic etiology of Alzheimer’s disease and related dementias. *Nat Genet*. 2022;54(4):412-436. doi:10.1038/s41588-022-01024-z

5. Kim JJ, Vitale D, Otani DV, et al. Multi-ancestry genome-wide association meta-analysis of Parkinson’s disease. *Nat Genet*. 2024;56(1):27-36. doi:10.1038/s41588-023-01584-8

6. Nalls MA, Blauwendraat C, Vallerga CL, et al. Identification of novel risk loci, causal insights, and heritable risk for Parkinson’s disease: a meta-analysis of genome-wide association studies. *Lancet Neurol*. 2019;18(12):1091-1102. doi:10.1016/S1474-4422(19)30320-5

7. Ferrari R, Hernandez DG, Nalls MA, et al. Frontotemporal dementia and its subtypes: a genome-wide association study. *Lancet Neurol*. 2014;13(7):686-699. doi:10.1016/S1474-4422(14)70065-1
