# Supplementary material for: Genetic and multi-omic resources for Alzheimer disease and related dementia from the Knight Alzheimer Disease Research Center
Source: Sci Data. 2024 Jul 12;11:768. doi: 10.1038/s41597-024-03485-9 (PMC11245521; doi:10.1038/s41597-024-03485-9)
Supplement: Supplementary file 3 — Appendix 3 [file 41597_2024_3485_MOESM3_ESM.docx]

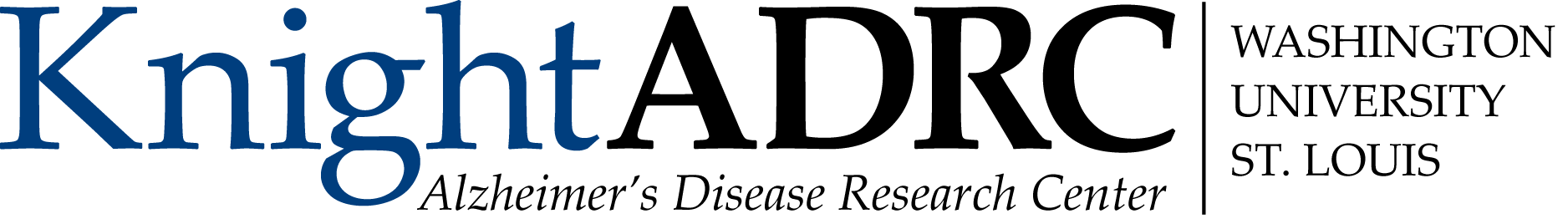


CSF SomaScan 7K Proteomic Data Methods

Jigyasha Timsina^1,2^, Yun Ju Sung^1,2,3^, Carlos Cruchaga^1,2,4^

^1^Department of Psychiatry, Washington University School of Medicine, St. Louis, MO, USA

^2^Neurogenomics and Informatics Center, Washington University School of Medicine, St. Louis, MO, USA

^3^Division of Biostatistics, Washington University School of Medicine, St. Louis, MO, USA

^4^Hope Center for Neurologic Diseases, Washington University, St. Louis, MO, USA

Table of Contents

[Introduction 3](#_Toc164416257)

[Summary 3](#_Toc164416258)

[Methodology 3](#_Toc164416259)

[Dataset information 8](#_Toc164416260)

[References 8](#_Toc164416261)

[About the Authors 9](#_Toc164416262)

# Introduction

SomaScan 7K (v4.1) was used to measure CSF proteomic levels of approximately 7000 proteins.

# Summary

Numerous CSF proteomic biomarkers have been identified for AD (Sung et al., 2023). Extensive GWAS analyses have highlighted 75 risk loci. Amyloid/tau pathways and microglia implication were enriched among these loci (Bellenguez et al., 2022). To link the AD genetic risk loci to their modulated functional molecules, protein quantitative trait loci (pQTL) for CSF, plasma and brain proteins have been investigated extensively (Yang et al., 2021). In this project, SomaScan 7K platform was applied to measure the CSF proteins.

# Methodology

Total 7584 analytes across 3065 samples from 5 cohorts including Knight ADRC (N=948) were assayed using SomaLogic’s SomaScan platform at once.Knight ADRC cohort was QCed together with 4 other cohorts.

Samples from the 5 cohorts were received and stored at -80°C. These samples were checked against their incoming manifests, confirming tube label and cryobox location. Samples were in multiple types of tubes, so similar tube types were grouped together by cohort to facilitate the transfer to 2D-barcoded tubes. Within each cohort, samples were arranged by available volume. All tube movement was done on dry ice to ensure that the samples did not thaw as they were rearranged. The afternoon prior to aliquoting, samples were moved from the cryoboxes to 96-well plastic racks, which were placed on wet ice in a refrigerator set to 4°C. Samples were then thawed for aliquoting overnight for approximately 14 hours, to allow for even thawing and prevent any part of the sample from warming above 4°C. The following morning, the samples were moved from the plastic racks to Corning CoolRack modules pre-chilled at 4°C. The final thawing stage was 30-35 minutes.

Samples were transferred one rack (96 samples) at a time. A separate paper checklist was completed for each rack of 96 samples. Before aliquoting samples, the plating manifest was compared with source tube label and location in the cold rack, and the 2D barcoded destination tubes were scanned. The time-stamped, tube scan file and photo of the source tube lids were moved to a unique folder on WUSTL Box.

Just before the aliquoting began, samples were centrifuged for 10 secs @1000g. Transfers were performed one column (8 samples) at a time, using an 8-channel repeating pipette. Samples were moved into the corresponding column in a second cold rack, and then opened. Samples were transferred into the corresponding column on the destination plates. Source tubes were closed and destination tubes were capped. This process was repeated until all 96 source samples were aliquoted into four destination plates. This project used tubes from the third destination plate. A post-scan of the 2D-barcoded tubes was conducted, and samples were flash frozen on dry ice for 15 minutes. A post-photo of the source rack was stored on WUSTL Box along with the checklist.

***Randomization of samples across plates***

We performed randomization check of samples across plates using ANOVA and Chi-squared test as applicable. We did not find batch effect among the plates.


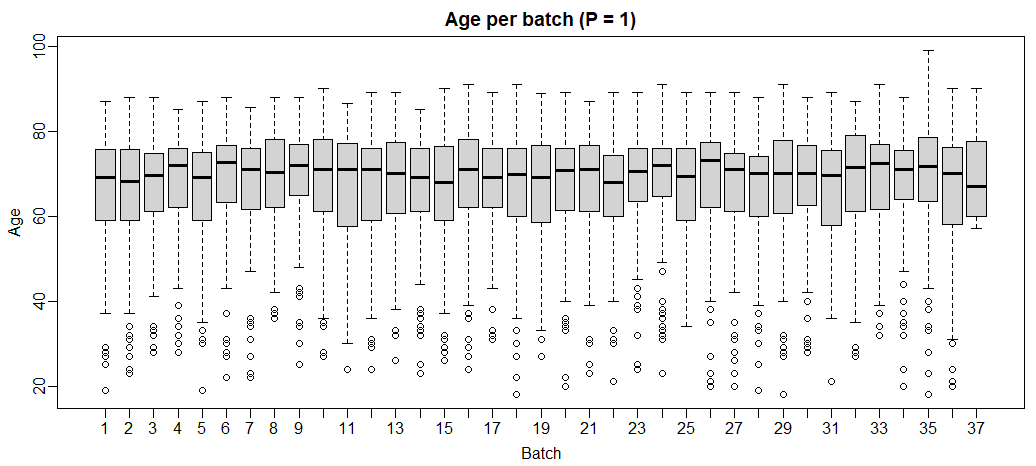


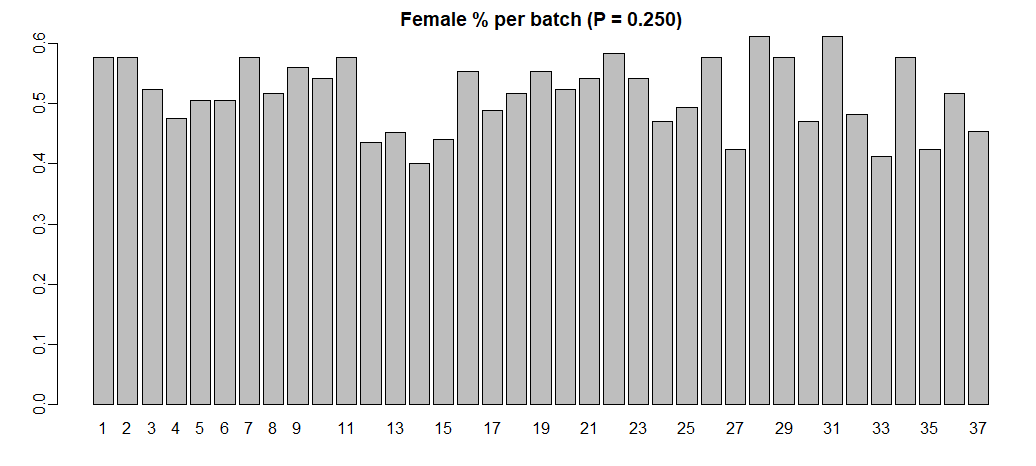


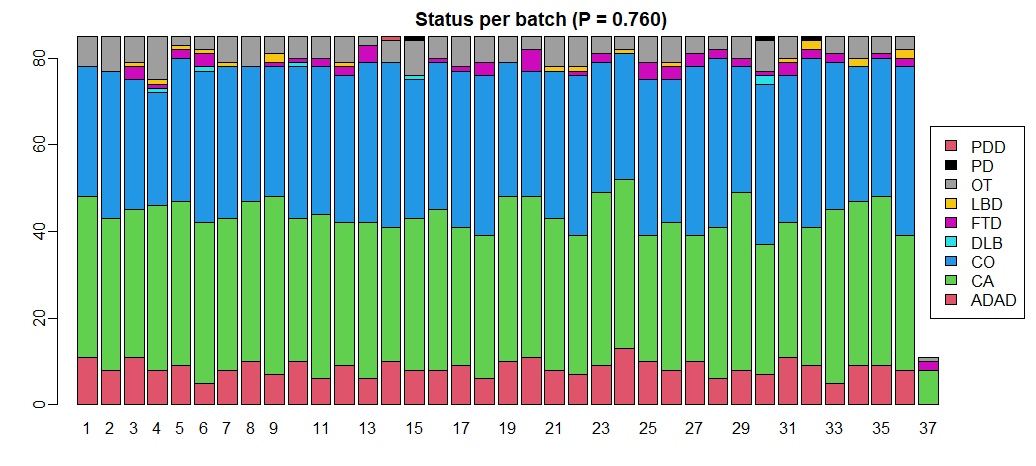


Figure 1: Randomization of samples across plates did not find difference of age, sex and disease status across plates. The corresponding P-values from each analysis is included in plot title. ADAD, Autosomal Dominant Alzheimer's Disease; CA, Alzheimer’s Disease Cases; CO, Healthy Controls; DLB, Dementia with Lewy bodies; FTD, Frontotemporal Dementia; LBD, Lewy Body Dementia; OT, Others; PD, Parkinson's disease; PDD, Parkinson’s Disease Dementia.

***Protein Measurement and Initial Data Normalization***

The protein levels were reported as Relative Fluorescence Unit (RFU). All data normalization steps were performed by SomaLogic. Briefly, Hybridization normalization was performed at the sample level. Aptamers were then divided into three normalization groups: S1, S2 and S3; based on the observed signal to noise ratio in technical replicates and samples. This division was done to avoid combining features with different level of protein signal for additional normalization steps(Timsina et al., 2022). Median normalization was then performed to remove other assay biases such as protein concentration, pipetting variation, variation in reagent concentrations, and assay timing among others (Candia et al., 2017; Gold et al., 2010). Finally, normalization to a reference was performed on individual samples to account for additional technical variance. This normalization step was performed using iterative Adaptive Normalization by Maximum Likelihood (ANML), a modification of median normalization.

***Correlation of duplicated samples***

Two samples, from one of the other cohorts measured in the complete data matrix, were from the same individual. The correlation of the two samples in raw RFU and log 10 (RFU) was plotted by all analytes, S1 analytes, S2 analytes and S3 analytes. Overall, a very high correlation between both measures was observed.

Figure 2: Correlation of duplicated samples among all analytes (A), S1 analytes (B), S2 analytes (C) and S3 analytes (D). Correlation Coefficient > 0.9 for all comparisons.

***QC pipeline overview***

All cohorts are QCed together without any cohort wise stratification. Somalogic’s proprietary .adat file contains the expression matrix for both samples and non-samples (Buffer, Calibrators and QC controls) are in the same file. For our QC, we divide these into two separate matrixes for ease of manipulation. Further quality control was performed on the normalized SOMAscan7k data provided by SomaLogic using in-house protocol. Aptamers were removed if they failed either of two criteria: first, if the maximum absolute difference between aptamer scale factor and median scale factor of any plate is >= 0.5; second, if the median cross-plate coefficient of variation (CV) was > = 0.15. Interquartile range (IQR) was then calculated for every aptamer based on log-10 transformed aptamer levels. Aptamer values outside of 1.5-fold of the IQR were replaced with NA values. Aptamers with call rate <65% (aptamer measurement in less than 65% of samples) were excluded, and the same criteria was used to remove samples. Call rate for aptamers was then recalculated and a more stringent call rate threshold of 85% was applied. Sample call rate was recalculated after aptamer removal and a call rate threshold of 85% was applied at the sample level. The flowchart below shows the steps applied during QC and the resulting number of samples and analytes at each step. A subset of the final matrix for Knight ADRC samples only were extracted for data sharing purposes.

**NOTE:** Apart from the analytes targeting human proteins, 2 other analytes targeting HIV were retained in the final matrix. A subset of the final matrix for Knight ADRC samples only were extracted for data sharing purposes.

# Dataset information

Final data shared with Knight ADRC are after our QC procedure and in the RFU units.

# References

Bellenguez, C., Küçükali, F., Jansen, I. E., Kleineidam, L., Moreno-Grau, S., Amin, N., Naj, A. C., Campos-Martin, R., Grenier-Boley, B., Andrade, V., Holmans, P. A., Boland, A., Damotte, V., van der Lee, S. J., Costa, M. R., Kuulasmaa, T., Yang, Q., de Rojas, I., Bis, J. C., … Lambert, J. C. (2022). New insights into the genetic etiology of Alzheimer’s disease and related dementias. *Nature Genetics 2022 54:4*, *54*(4), 412–436. https://doi.org/10.1038/s41588-022-01024-z

Candia, J., Cheung, F., Kotliarov, Y., Fantoni, G., Sellers, B., Griesman, T., Huang, J., Stuccio, S., Zingone, A., Ryan, B. M., Tsang, J. S., & Biancotto, A. (2017). Assessment of Variability in the SOMAscan Assay. *Scientific Reports 2017 7:1*, *7*(1), 1–13. https://doi.org/10.1038/s41598-017-14755-5

Gold, L., Ayers, D., Bertino, J., Bock, C., Bock, A., Brody, E. N., Carter, J., Dalby, A. B., Eaton, B. E., Fitzwater, T., Flather, D., Forbes, A., Foreman, T., Fowler, C., Gawande, B., Goss, M., Gunn, M., Gupta, S., Halladay, D., … Zichi, D. (2010). Aptamer-Based Multiplexed Proteomic Technology for Biomarker Discovery. *PLOS ONE*, *5*(12), e15004. https://doi.org/10.1371/JOURNAL.PONE.0015004

Sung, Y. J., Yang, C., Norton, J., Johnson, M., Fagan, A., Bateman, R. J., Perrin, R. J., Morris, J. C., Farlow, M. R., Chhatwal, J. P., Schofield, P. R., Chui, H., Wang, F., Novotny, B., Eteleeb, A., Karch, C., Schindler, S. E., Rhinn, H., Johnson, E. C. B., … Cruchaga, C. (2023). Proteomics of brain, CSF, and plasma identifies molecular signatures for distinguishing sporadic and genetic Alzheimer’s disease. *Science Translational Medicine*, *15*(703). https://doi.org/10.1126/SCITRANSLMED.ABQ5923/SUPPL_FILE/SCITRANSLMED.ABQ5923_MDAR_REPRODUCIBILITY_CHECKLIST.PDF

Timsina, J., Gomez-Fonseca, D., Wang, L., Do, A., Western, D., Alvarez, I., Aguilar, M., Pastor, P., Henson, R. L., Herries, E., Xiong, C., Schindler, S. E., Fagan, A. M., Bateman, R. J., Farlow, M., Morris, J. C., Perrin, R., Moulder, K., Hassenstab, J., … Cruchaga, C. (2022). Comparative analysis of Alzheimer’s disease Cerebrospinal fluid biomarkers measurement by multiplex SOMAscan platform and immunoassay-based approach. *Journal of Alzheimer’s Disease : JAD*, *89*(1), 193. https://doi.org/10.3233/JAD-220399

Yang, C., Farias, F. H. G., Ibanez, L., Suhy, A., Sadler, B., Fernandez, M. V., Wang, F., Bradley, J. L., Eiffert, B., Bahena, J. A., Budde, J. P., Li, Z., Dube, U., Sung, Y. J., Mihindukulasuriya, K. A., Morris, J. C., Fagan, A. M., Perrin, R. J., Benitez, B. A., … Cruchaga, C. (2021). Genomic atlas of the proteome from brain, CSF and plasma prioritizes proteins implicated in neurological disorders. *Nature Neuroscience*, *24*(9), 1302–1312. https://doi.org/10.1038/S41593-021-00886-6

# About the Authors

This document was prepared by Jigyasha Timsina, Yun Ju Sung, Carlos Cruchaga. For more information, please contact Jigyasha Timsina at [timsinaj@wustl.edu](mailto:timsinaj@wustl.edu), Yun Ju Sung at [yunju@wustl.edu](mailto:yunju@wustl.edu), Carlos Cruchaga at cruchagac@wustl.edu.
