# Supplementary material for: Genetic and multi-omic resources for Alzheimer disease and related dementia from the Knight Alzheimer Disease Research Center
Source: Sci Data. 2024 Jul 12;11:768. doi: 10.1038/s41597-024-03485-9 (PMC11245521; doi:10.1038/s41597-024-03485-9)
Supplement: Supplementary file 4 — Appendix 4 [file 41597_2024_3485_MOESM4_ESM.docx]

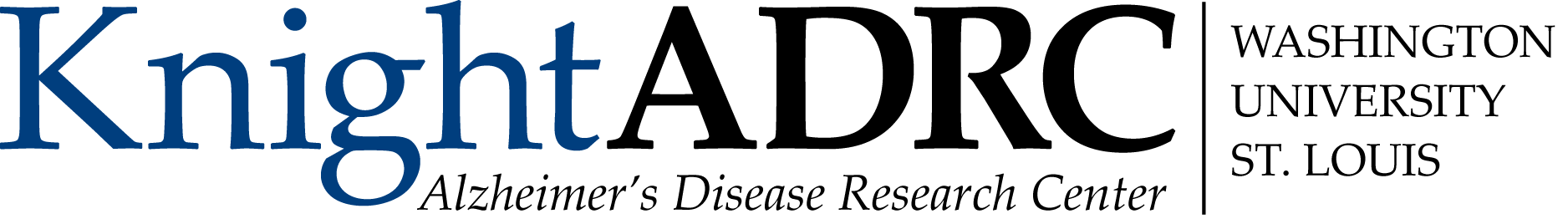


Plasma SomaScan 7K Proteomic Data Methods

Jigyasha Timsina^1,2^, Yun Ju Sung^1,2,3^, Carlos Cruchaga^1,2,4^

^1^Department of Psychiatry, Washington University School of Medicine, St. Louis, MO, USA

^2^Neurogenomics and Informatics Center, Washington University School of Medicine, St. Louis, MO, USA

^3^Division of Biostatistics, Washington University School of Medicine, St. Louis, MO, USA

^4^Hope Center for Neurologic Diseases, Washington University, St. Louis, MO, USA

Table of Contents

[Introduction 3](#_Toc164427754)

[Summary 3](#_Toc164427755)

[Methodology 3](#_Toc164427756)

[Dataset information 8](#_Toc164427757)

[References 8](#_Toc164427758)

[About the Authors 8](#_Toc164427759)

# Introduction

SomaScan 7K (v4.1) was used to measure Plasma proteomic levels of approximately 7000 proteins.

# Summary

In recent years, plasma has gained popularity as an alternative to CSF in AD biomarker research due to its ease of access and comparatively lower risk of complications(Blennow et al., 2012). Besides, with advancement of technology, newer proteomic platforms, such as SomaScan, have been shown to effectively capture changes in blood reflective of brain disorders for other neurological conditions (Posavi et al., 2019; Shi et al., 2019). In this project, SomaScan 7K platform was applied to measure the plasma proteins.

# Methodology

Total 7584 analytes across 3229 samples from 2 cohorts including Knight ADRC (N=3170) were assayed using SomaLogic’s SomaScan platform at once. Knight ADRC cohort was QCed together with the other cohort.

Non- fasted blood samples were collected at the visit time, immediately centrifuged, and stored at − 80°C. Samples were transferred one rack (96 samples) at a time. A separate paper checklist was completed for each rack of 96 samples. Before aliquoting samples, the plating manifest was compared with source tube label and location in the cold rack, and the 2D barcoded destination tubes were scanned. The time-stamped, tube scan file and photo of the source tube lids were moved to a unique folder on WUSTL Box. A post-scan of the 2D-barcoded tubes was conducted. A post-photo of the source rack was stored on WUSTL Box along with the checklist.

***Randomization of samples across plates***

We performed randomization check of samples across plates using ANOVA and Chi-squared test as applicable. We did not find batch effect among the plates.


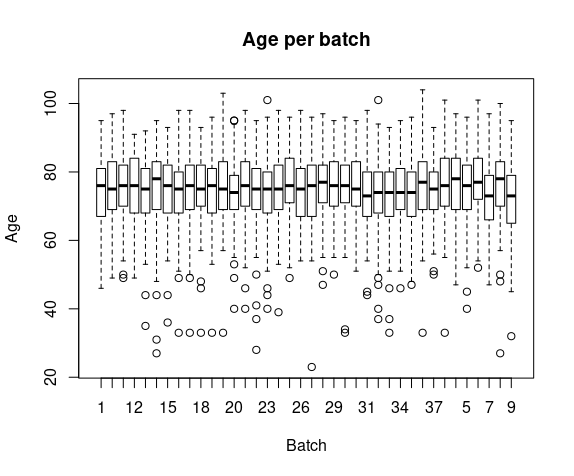


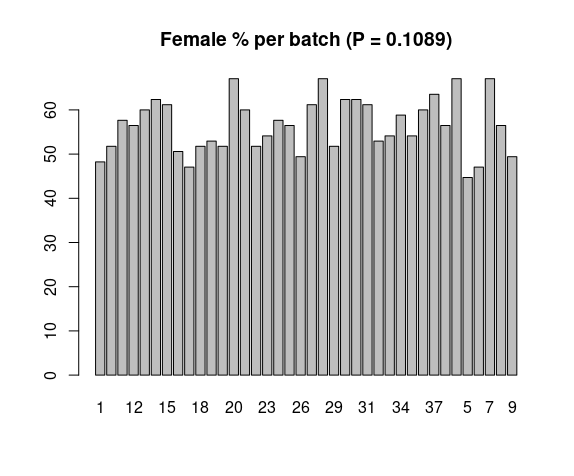


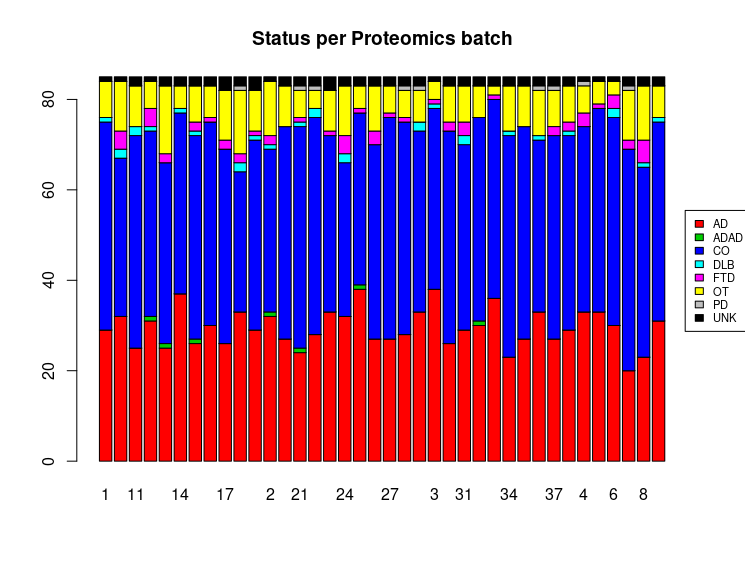


Figure 1: Randomization of samples across plates did not find difference of age, sex and disease status across plates. P-value 0.284, 0.109 and 0.96 found for age, gender and status respectively [ANOVA and Chi-Square as applicable]. ADAD, Autosomal Dominant Alzheimer's Disease; AD, Alzheimer’s Disease Cases; CO, Healthy Controls; DLB, Dementia with Lewy bodies; FTD, Frontotemporal Dementia; OT, Others; PD, Parkinson's disease; UNK, Unknown at the time of sample selection.

***Protein Measurement and Initial Data Normalization***

The protein levels were reported as Relative Fluorescence Unit (RFU). All data normalization steps were performed by SomaLogic. Briefly, data were first normalized using hybridization controls to mitigate variation within the run that comes from the readout steps: transfer to Agilent slides, hybridization, wash, and scan etc. followed by median signal normalization across pooled calibrator replicates within the run to mitigate within-run technical variation. Median signal normalization is performed using Adaptive Normalization by Maximum Likelihood (ANML) for specimen types and studies shown to be consistent with pre- defined population references or, alternatively, using median normalization to a study- specific reference.

***QC pipeline overview***

Both cohorts were QCed together without any cohort wise stratification. Somalogic’s proprietary .adat file contains the expression matrix for both samples and non-samples (Buffer, Calibrators and QC controls) are in the same file. For our QC, we divide these into two separate matrixes for ease of manipulation. Further quality control was performed on the normalized SOMAscan7k data provided by SomaLogic using in-house protocol. Aptamers were removed if they failed either of three criteria: first, if the average expression of any analyte was outside 3SD of its average expression in buffer in more than 15% of the total samples; second, if the maximum absolute difference between aptamer scale factor and median scale factor of any plate is >= 0.5; third, if the median cross-plate coefficient of variation (CV) was > = 0.15. Interquartile range (IQR) was then calculated for every aptamer based on log-10 transformed aptamer levels. Aptamer values outside of 1.5-fold of the IQR were replaced with NA values. Aptamers with call rate <65% (aptamer measurement in less than 65% of samples) were excluded, and the same criteria was used to remove samples. Call rate for aptamers was then recalculated and a more stringent call rate threshold of 85% was applied. Sample call rate was recalculated after aptamer removal and a call rate threshold of 85% was applied at the sample level. The flowchart below shows the steps applied during QC and the resulting number of samples and analytes at each step. A subset of the final matrix for Knight ADRC samples only were extracted for data sharing purposes.

**NOTE:** Apart from the analytes targeting human proteins, 2 other analytes targeting HIV were retained in the final matrix. A subset of the final matrix for Knight ADRC samples only were extracted for data sharing purposes.

# Dataset information

Final data shared with Knight ADRC are after our QC procedure and in the RFU units.

# References

Blennow, K., Zetterberg, H., & Fagan, A. M. (2012). Fluid Biomarkers in Alzheimer Disease. *Cold Spring Harbor Perspectives in Medicine*, *2*(9). https://doi.org/10.1101/CSHPERSPECT.A006221

Posavi, M., Diaz-Ortiz, M., Liu, B., Swanson, C. R., Skrinak, R. T., Hernandez-Con, P., Amado, D. A., Fullard, M., Rick, J., Siderowf, A., Weintraub, D., McCluskey, L., Trojanowski, J. Q., Dewey, R. B., Huang, X., & Chen-Plotkin, A. S. (2019). Characterization of Parkinson’s disease using blood-based biomarkers: A multicohort proteomic analysis. *PLoS Medicine*, *16*(10). https://doi.org/10.1371/JOURNAL.PMED.1002931

Shi, L., Westwood, S., Baird, A. L., Winchester, L., Dobricic, V., Kilpert, F., Hong, S., Franke, A., Hye, A., Ashton, N. J., Morgan, A. R., Bos, I., Vos, S. J. B., Buckley, N. J., Kate, M. ten, Scheltens, P., Vandenberghe, R., Gabel, S., Meersmans, K., … Nevado-Holgado, A. J. (2019). Discovery and validation of plasma proteomic biomarkers relating to brain amyloid burden by SOMAscan assay. *Alzheimer’s & Dementia*, *15*(11), 1478. https://doi.org/10.1016/J.JALZ.2019.06.4951

# About the Authors

This document was prepared by Jigyasha Timsina, Yun Ju Sung, Carlos Cruchaga. For more information, please contact Jigyasha Timsina at [timsinaj@wustl.edu](mailto:timsinaj@wustl.edu), Yun Ju Sung at [yunju@wustl.edu](mailto:yunju@wustl.edu), Carlos Cruchaga at cruchagac@wustl.edu.
