# Supplementary material for: Genetic and multi-omic resources for Alzheimer disease and related dementia from the Knight Alzheimer Disease Research Center
Source: Sci Data. 2024 Jul 12;11:768. doi: 10.1038/s41597-024-03485-9 (PMC11245521; doi:10.1038/s41597-024-03485-9)
Supplement: Supplementary file 5 — Appendix 5 [file 41597_2024_3485_MOESM5_ESM.docx]

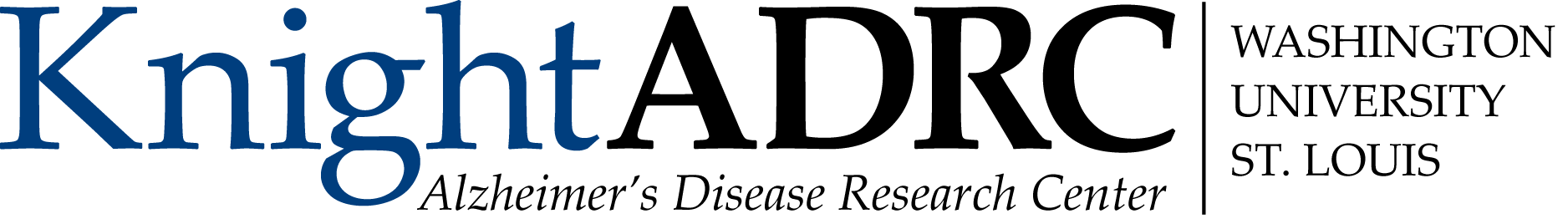


CSF Metabolon HD4 Data Methods

Jigyasha Timsina^1,2^, Ciyang Wang^1,2^, Yun Ju Sung^1,2,3^, Carlos Cruchaga^1,2,4^

^1^Department of Psychiatry, Washington University School of Medicine, St. Louis, MO, USA

^2^NeuroGenomics and Informatics Center, Washington University School of Medicine, St. Louis, MO, USA

^3^Division of Biostatistics, Washington University School of Medicine, St. Louis, MO, USA

^4^Hope Center for Neurologic Diseases, Washington University, St. Louis, MO, USA

# Introduction

Metabolon HD4 was used to measure CSF metabolites levels.

# Summary

Numerous CSF metabolites biomarkers have been identified for AD. Extensive GWAS analyses have highlighted 75 risk loci. Amyloid/tau pathways and microglia implication were enriched among these loci (Bellenguez et al., 2022). Previous metabolome wide studies have highlighted metabolite associations significant in AD (Panyard et al., 2021). In this project, Metabolon HD4 platform were applied to measure the CSF metabolites.

# Methodology

A total of 2987 cerebral spinal fluid (CSF) samples from 5 cohorts, including Knight ADRC were profiled by Metabolon. The Knight ADRC cohort (N=948) were QCed together with the other 4 cohorts.

Samples from the 5 cohorts were received and stored at -80°C. These samples were checked against their incoming manifests, confirming tube label and cryobox location. Samples were in multiple types of tubes, so similar tube types were grouped together by cohort to facilitate the transfer to 2D-barcoded tubes. Within each cohort, samples were arranged by available volume. All tube movement was done on dry ice to ensure that the samples did not thaw as they were rearranged. The afternoon prior to aliquoting, samples were moved from the cryoboxes to 96-well plastic racks, which were placed on wet ice in a refrigerator set to 4°C. Samples were then thawed for aliquoting overnight for approximately 14 hours, to allow for even thawing and prevent any part of the sample from warming above 4°C. The following morning, the samples were moved from the plastic racks to Corning CoolRack modules pre-chilled at 4°C. The final thawing stage was 30-35 minutes.

Samples were transferred one rack (96 samples) at a time. A separate paper checklist was completed for each rack of 96 samples. Before aliquoting samples, the plating manifest was compared with source tube label and location in the cold rack, and the 2D barcoded destination tubes were scanned. The time-stamped, tube scan file and photo of the source tube lids were moved to a unique folder on WUSTL Box.

Just before the aliquoting began, samples were centrifuged for 10 secs @1000g. Transfers were performed one column (8 samples) at a time, using an 8-channel repeating pipette. Samples were moved into the corresponding column in a second cold rack, and then opened. Samples were transferred into the corresponding column on the destination plates. Source tubes were closed and destination tubes were capped. This process was repeated until all 96 source samples were aliquoted into four destination plates. This project used tubes from the third destination plate. A post-scan of the 2D-barcoded tubes was conducted, and samples were flash frozen on dry ice for 15 minutes. A post-photo of the source rack was stored on WUSTL Box along with the checklist.

***Randomization of samples across plates***

We performed randomization check of samples across plates using ANOVA and Chi-squared test as applicable. We did not find batch effect among the plates.


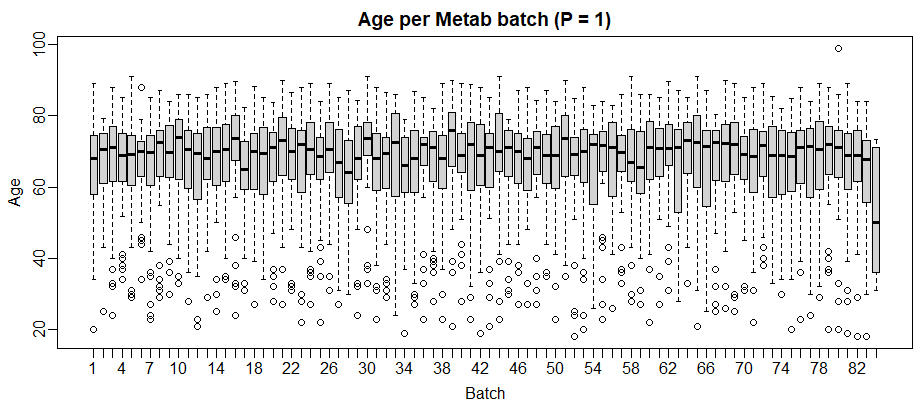


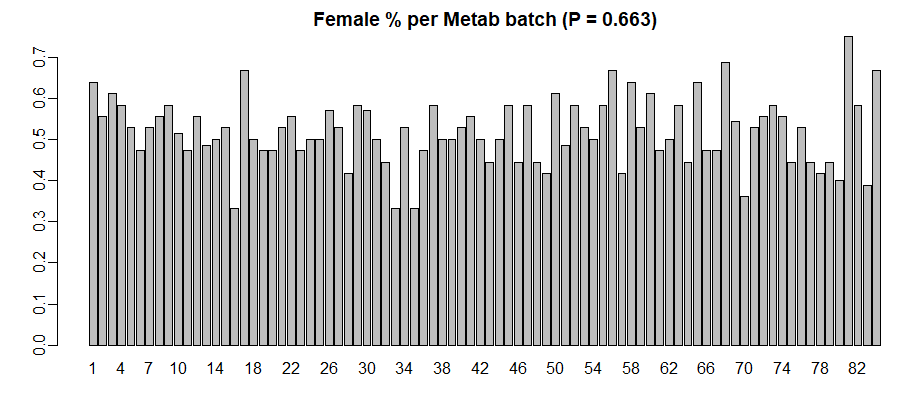


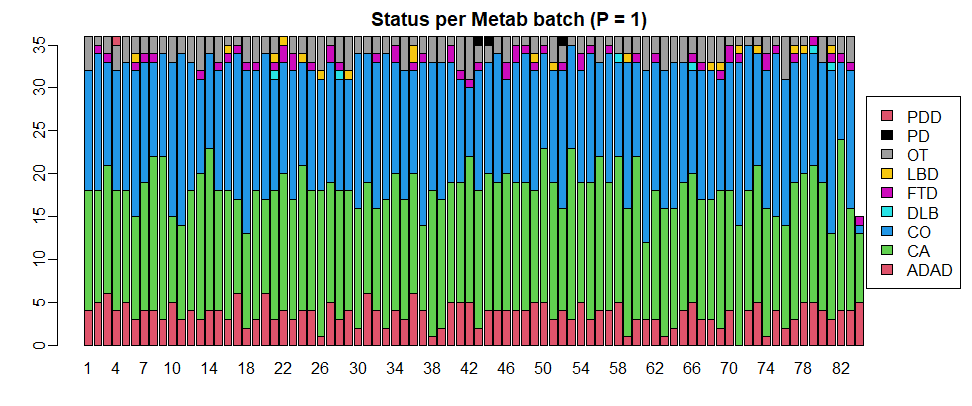


Figure 1: Randomization of samples across plates did not find difference of age, sex and disease status across plates. ADAD, Autosomal Dominant Alzheimer's Disease; CA, Alzheimer’s Disease Cases; CO, Healthy Controls; DLB, Dementia with Lewy bodies; FTD, Frontotemporal Dementia; LBD, Lewy Body Dementia; OT, Others; PD, Parkinson's disease; PDD, Parkinson’s Disease Dementia. The corresponding P-values from each analysis is included in plot title.

***Metabolite Measurement:***

A total of 478 metabolites were measured using HD4 Metabolon Platform. Metabolites were quantified using ultrahigh performance liquid chromatography-tandem mass spectrometry (UPLC-MS/MS) (Metabolon Precision Metabolomics platform).

***QC pipeline overview:***

All cohorts were QCed together without any cohort wise stratification. The flowchart below shows the steps applied during QC and the resulting number of samples and analytes at each step. The initial steps of quality control assessed the missingness of each sample and each metabolite. First, a sample with > 50% missingness was removed. As a next step, metabolites were filtered based on missingness. Metabolites were defined by Metabolon to be either innate or foreign to human system as non-xenobiotics and xenobiotics respectively. Non-xenobiotics are expected to be present in many samples, while xenobiotics can be largely missing due to their foreign nature. Therefore, only non-xenobiotics with > 80% missingness were removed and xenobiotics were not assessed at this step. Due to the mixture of individual disease status (Control, AD, PD, FTD, aging) in all cohorts, we checked if the missingness of the removed metabolites could be due to some biological effect. To this end, we applied fisher’s exact tests and linear regression comparing each disease status group versus control group. However, none of the removed metabolites were recovered because we did not see any association between the metabolites and disease status. We then performed imputation for non-xenobiotics using minimum value of the metabolite, while xenobiotics were not imputed. Log10 transformation was applied to achieve approximate normal distribution. Next, given that metabolites with little variation throughout samples are non-informative for analysis, we removed metabolites that either had IQR equal to zero, or variance < 0.001. Following the non-informative metabolite removal, outlier detection was performed where in any metabolite level outside the range of values from the first quantile minus 1.5-fold IQR to the third quantile plus 1.5-fold IQR were marked as outliers. In addition, we removed metabolites with an overall limited number of values (N < 50) to ensure a sufficient power for analysis. Lastly, samples outliers, defined by > 5 std from the mean of principle component one or two, were excluded. A subset of the final matrix for Knight ADRC samples only were extracted for data sharing purposes.

**Note:** A subset of the final matrix for Knight ADRC samples only were extracted for data sharing purposes.

***Dataset information***

Final data shared with Knight ADRC are after our cleaning procedure and in the raw units transformed back from Log10 scale. The initial missing data points for non-Xenobiotic groups of metabolites have been imputed with minimum levels and the missing points currently in the dataset are due to result of our outlier detection step.

**References**

Bellenguez, C., Küçükali, F., Jansen, I. E., Kleineidam, L., Moreno-Grau, S., Amin, N., Naj, A. C., Campos-Martin, R., Grenier-Boley, B., Andrade, V., Holmans, P. A., Boland, A., Damotte, V., van der Lee, S. J., Costa, M. R., Kuulasmaa, T., Yang, Q., de Rojas, I., Bis, J. C., … Lambert, J. C. (2022). New insights into the genetic etiology of Alzheimer’s disease and related dementias. *Nature Genetics 2022 54:4*, *54*(4), 412–436. https://doi.org/10.1038/s41588-022-01024-z

Panyard, D. J., Kim, K. M., Darst, B. F., Deming, Y. K., Zhong, X., Wu, Y., Kang, H., Carlsson, C. M., Johnson, S. C., Asthana, S., Engelman, C. D., & Lu, Q. (2021). Cerebrospinal fluid metabolomics identifies 19 brain-related phenotype associations. *Communications Biology 2021 4:1*, *4*(1), 1–11. https://doi.org/10.1038/s42003-020-01583-z

# About the Authors

This document was prepared by Jigyasha Timsina, Ciyang Wang, Yun Ju Sung, Carlos Cruchaga. For more information please contact Jigyasha Timsina by email at [timsinaj@wustl.edu](mailto:timsinaj@wustl.edu), Ciyang Wang by email at [wangciyang@wustl.edu](mailto:wangciyang@wustl.edu), Yun Ju Sung at [yunju@wustl.edu](mailto:yunju@wustl.edu), Carlos Cruchaga at cruchagac@wustl.edu.
