# Supplementary material for: Genetic and multi-omic resources for Alzheimer disease and related dementia from the Knight Alzheimer Disease Research Center
Source: Sci Data. 2024 Jul 12;11:768. doi: 10.1038/s41597-024-03485-9 (PMC11245521; doi:10.1038/s41597-024-03485-9)
Supplement: Supplementary file 6 — Appendix 6 [file 41597_2024_3485_MOESM6_ESM.docx]

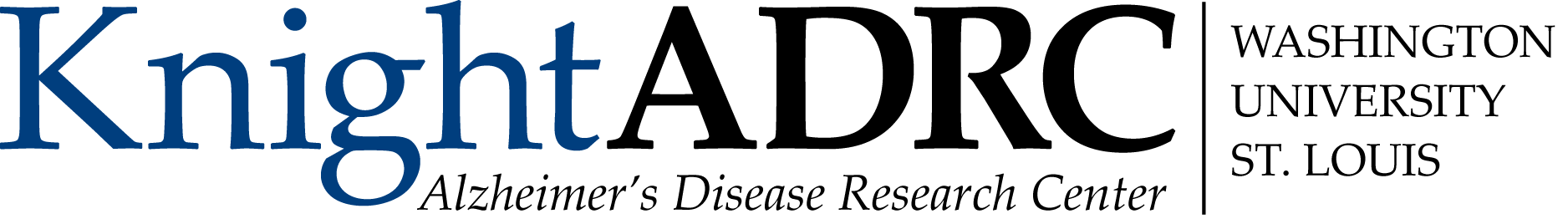


Plasma Metabolon HD4 Data Methods

Jigyasha Timsina^1,2^, Ciyang Wang^1,2^, Yun Ju Sung^1,2,3^, Carlos Cruchaga^1,2,4^

^1^Department of Psychiatry, Washington University School of Medicine, St. Louis, MO, USA

^2^NeuroGenomics and Informatics Center, Washington University School of Medicine, St. Louis, MO, USA

^3^Division of Biostatistics, Washington University School of Medicine, St. Louis, MO, USA

^4^Hope Center for Neurologic Diseases, Washington University, St. Louis, MO, USA

# Introduction

Metabolon HD4 was used to measure Plasma metabolites levels.

# Summary

In recent years, plasma has gained popularity as an alternative to CSF in AD biomarker research due to its ease of access and comparatively lower risk of complications (Blennow et al., 2012). Previous metabolome wide studies have highlighted metabolite associations significant in AD (Panyard et al., 2021). In this project, Metabolon HD4 platform were applied to measure the plasma metabolites.

# Methodology

A total of 3170 plasma samples from Knight ADRC cohort were profiled by Metabolon.

Non- fasted blood samples were collected at the visit time, immediately centrifuged, and stored at − 80°C. Samples were transferred one rack (96 samples) at a time. A separate paper checklist was completed for each rack of 96 samples. Before aliquoting samples, the plating manifest was compared with source tube label and location in the cold rack, and the 2D barcoded destination tubes were scanned. The time-stamped, tube scan file and photo of the source tube lids were moved to a unique folder on WUSTL Box. A post-scan of the 2D-barcoded tubes was conducted. A post-photo of the source rack was stored on WUSTL Box along with the checklist.

***Randomization of samples across plates***

We performed randomization check of samples across plates using ANOVA and Chi-squared test as applicable. We did not find batch effect among the plates.


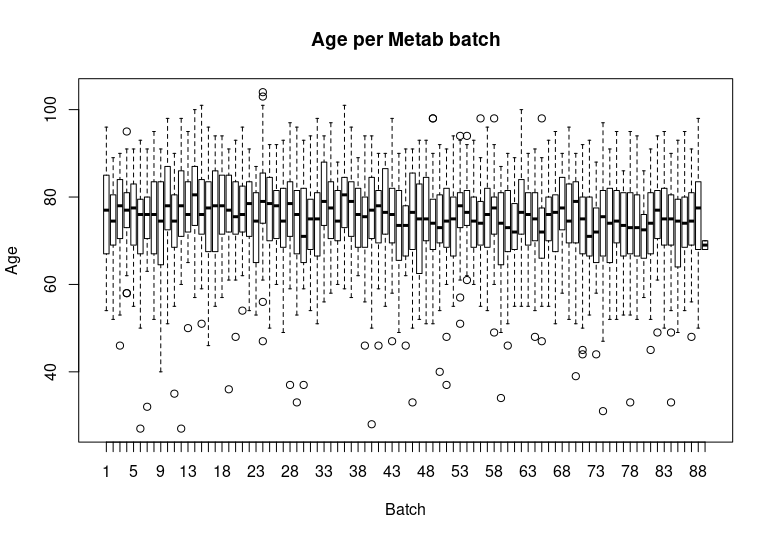


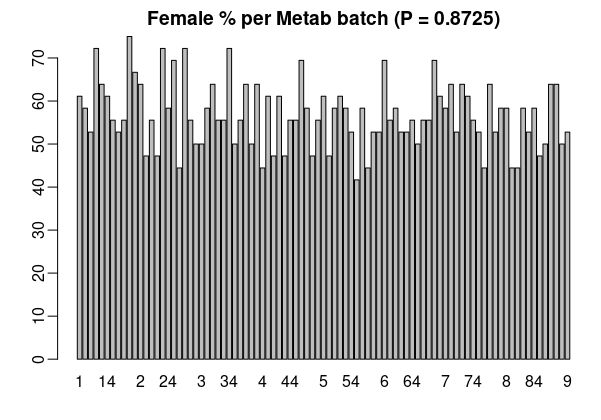


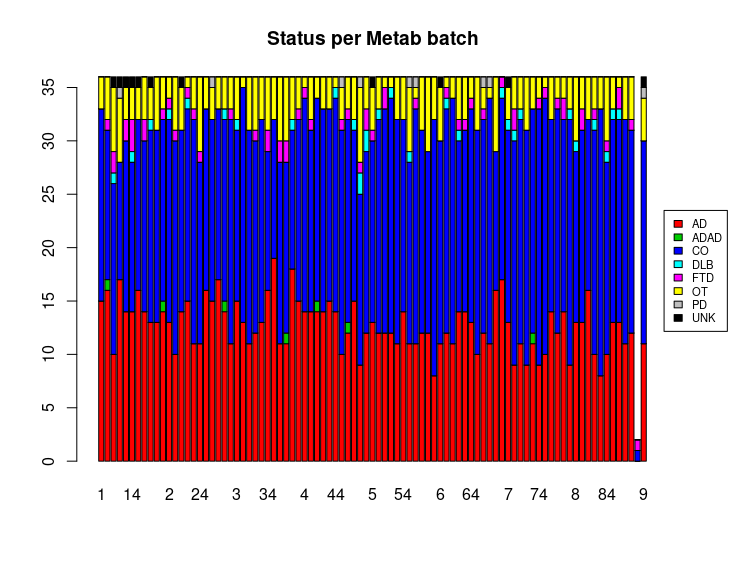


Figure 1: Randomization of samples across plates did not find difference of age, sex and disease status across plates. P-value 0.07, 0.8 and 0.97 found for age, gender and status respectively [ANOVA and Chi-Square as applicable]. ADAD, Autosomal Dominant Alzheimer's Disease; CA, Alzheimer’s Disease Cases; CO, Healthy Controls; DLB, Dementia with Lewy bodies; FTD, Frontotemporal Dementia; LBD, Lewy Body Dementia; OT, Others; PD, Parkinson's disease; PDD, Parkinson’s Disease Dementia. The corresponding P-values from each analysis is included in plot title.

***Metabolite Measurement:***

A total of 1718 metabolites were measured using HD4 Metabolon Platform. Metabolites were quantified using ultrahigh performance liquid chromatography-tandem mass spectrometry (UPLC-MS/MS) (Metabolon Precision Metabolomics platform).

***QC pipeline overview:***

The flowchart below shows the steps applied during QC and the resulting number of samples and analytes at each step. The initial steps of quality control assessed the missingness of each sample and each metabolite. First, a sample with > 50% missingness was removed. As a next step, metabolites were filtered based on missingness. Metabolites were defined by Metabolon to be either innate or foreign to human system as non-xenobiotics and xenobiotics respectively. Non-xenobiotics are expected to be present in many samples, while xenobiotics can be largely missing due to their foreign nature. Therefore, only non-xenobiotics with > 80% missingness were removed and xenobiotics were not assessed at this step. Due to the mixture of individual disease status (Control, AD, PD, FTD, aging) in all cohorts, we checked if the missingness of the removed metabolites could be due to some biological effect. To this end, we applied fisher’s exact tests and linear regression comparing each disease status group versus control group. However, none of the removed metabolites were recovered because we did not see any association between the metabolites and disease status. We then performed imputation for non-xenobiotics using minimum value of the metabolite, while xenobiotics were not imputed. Log10 transformation was applied to achieve approximate normal distribution. Next, given that metabolites with little variation throughout samples are non-informative for analysis, we removed metabolites that either had IQR equal to zero, or variance < 0.001. Following the non-informative metabolite removal, outlier detection was performed where in any metabolite level outside the range of values from the first quantile minus 1.5-fold IQR to the third quantile plus 1.5-fold IQR were considered to be outliers. In addition, we removed metabolites with an overall limited number of values (N < 50) to ensure a sufficient power for analysis. Lastly, samples outliers, defined by > 5 std from the mean of principle component one or two, were excluded. A subset of the final matrix for Knight ADRC samples only were extracted for data sharing purposes.

***Dataset information***

Final data shared with Knight ADRC are after our cleaning procedure and in the raw units transformed back from Log10 scale. The initial missing data points for non-Xenobiotic groups of metabolites have been imputed with minimum levels and the missing points currently in the dataset are due to result of our outlier detection step.

**References**

Blennow, K., Zetterberg, H., & Fagan, A. M. (2012). Fluid Biomarkers in Alzheimer Disease. *Cold Spring Harbor Perspectives in Medicine*, *2*(9). https://doi.org/10.1101/CSHPERSPECT.A006221

Panyard, D. J., Kim, K. M., Darst, B. F., Deming, Y. K., Zhong, X., Wu, Y., Kang, H., Carlsson, C. M., Johnson, S. C., Asthana, S., Engelman, C. D., & Lu, Q. (2021). Cerebrospinal fluid metabolomics identifies 19 brain-related phenotype associations. *Communications Biology 2021 4:1*, *4*(1), 1–11. https://doi.org/10.1038/s42003-020-01583-z

# About the Authors

This document was prepared by Jigyasha Timsina Ciyang Wang, Yun Ju Sung, Carlos Cruchaga. For more information please contact Jigyasha Timsina by email at [timsinaj@wustl.edu](mailto:timsinaj@wustl.edu), Ciyang Wang by email at [wangciyang@wustl.edu](mailto:wangciyang@wustl.edu), Yun Ju Sung at [yunju@wustl.edu](mailto:yunju@wustl.edu), Carlos Cruchaga at cruchagac@wustl.edu.
